# Machine learning model for intravenous immunoglobulin resistance in Kawasaki disease: model development and validation study

**DOI:** 10.1007/s12519-026-01075-w

**Published:** 2026-09-01

**Authors:** Jia-Ying Zhang, Ting-Jiao You, Jing Li, Jin-Feng Dong, Lei Xu, Xuan Li, Jun-Long Hu, Yun-Jia Tang, Miao Hou, Ying Liu, Zhen-Xing Xu, Hai-Tao Lv, Hong-Biao Huang

**Affiliations:** 1 Department of Cardiology, Children’s Hospital of Soochow University, Suzhou, China; 2 Institute of Pediatric Research, Children’s Hospital of Soochow University, Suzhou, China; 3 Department of Hematology, The First Affiliated Hospital of Fujian Medical University, Fuzhou, China; 4 Department of Hematology, National Regional Medical Center, Binhai Campus of the First Affliated Hospital, Fujian Medical University, Fuzhou, China; 5 Department of Pediatrics, Suzhou Municipal Hospital, The Affiliated Suzhou Hospital of Nanjing Medical University, Suzhou, China; 6 Department of Pediatrics, Pediatric Key Laboratory of Xiamen, The First Affiliated Hospital of Xiamen University, Xiamen, China; 7 Department of Pediatrics, The Affiliated Hospital of Yangzhou University, Yangzhou First People’s Hospital, Yangzhou, China; 8 Department of Pediatrics, Fujian Provincial Hospital, Fuzhou University Affiliated Provincial Hospital, 134 Dong Street, Fuzhou, China

**Keywords:** Artificial intelligence, Children, Intravenous immunoglobulin, Kawasaki disease, Machine learning, Pediatric, Risk assessment

## Abstract

**Background:**

Intravenous immunoglobulin (IVIG) resistance in Kawasaki disease (KD) increases coronary artery risk. Early prediction is crucial for improving outcomes. This study aimed to develop and validate a machine learning (ML) model for predicting IVIG resistance in children with KD.

**Methods:**

A retrospective cohort of patients with KD was used for model development, with external validation cohorts from Fuzhou and Yangzhou, and a prospective validation cohort. Clinical and laboratory variables were extracted from electronic medical records. We evaluated 12 algorithms and support vector machine (SVM) was selected for optimal performance. SHapley Additive exPlanations (SHAP) values assessed feature importance, followed by stepwise feature elimination. Model performance was evaluated using area under the receiver operating characteristic curve (AUC), calibration curves, and decision curve analysis (DCA). A web-based calculator was developed.

**Results:**

A total of 2371 patients with KD involved in the retrospective development cohort, 443 in Fuzhou cohort, 198 in Yangzhou cohort, and 253 in prospective validation cohort. The SVM model achieved AUCs of 0.782 in internal validation, 0.746 and 0.759 in the external validation cohorts from Fuzhou and Yangzhou, respectively, and 0.799 in prospective validation. The final model incorporated eight predictors, with SHAP analysis providing both global and local explanations of feature contributions. The model also demonstrated good calibration and favorable net benefit across clinically relevant thresholds in DCA.

**Conclusions:**

The SVM-based ML model using routine clinical data shows potential for predicting IVIG resistance in KD and may support early risk stratification.

**Graphical Abstract:**

**Figure:**
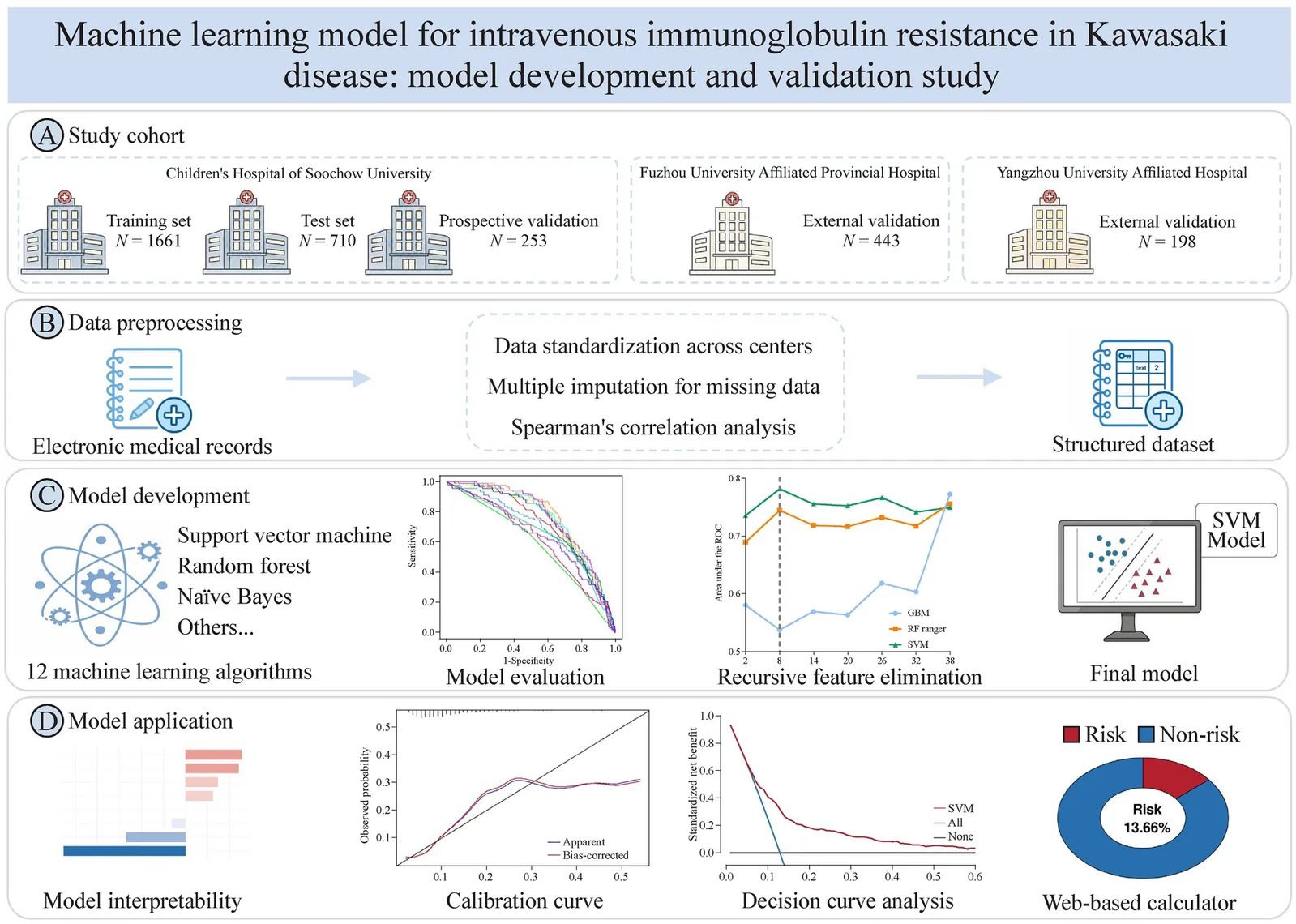

**Supplementary Information:**

The online version contains supplementary material available at https://doi.org/10.1007/s12519-026-01075-w.

## Introduction

Kawasaki disease (KD) is an acute febrile vasculitis predominantly affecting children and is the leading cause of acquired heart disease in developed countries [1]. Without timely treatment, approximately 20%–25% of patients develop coronary artery lesions (CALs), which may result in luminal narrowing, myocardial ischemia, and necrosis [2, 3]. High-dose intravenous immunoglobulin (IVIG) is the standard initial therapy. However, 10%–20% of patients are IVIG-resistant and remain at increased risk of severe cardiac complications [4]. Early identification of IVIG resistance is therefore essential, as intensified treatment strategies, including IVIG combined with corticosteroids or immunosuppressants, are recommended for high-risk patients [5]. To facilitate early risk stratification, several predictive models have been proposed, including the Egami, Kobayashi, Sano, and San Diego scoring systems [6–9]. While these models offer intuitive risk estimates, they rely on linear statistical approaches that often fail to accommodate complex, nonlinear interactions inherent in clinical datasets [10]. Moreover, existing prediction models are often constrained by factors such as limited sample sizes, potential bias from dichotomizing continuous variables, and insufficient internal or external validation, all of which may compromise both their generalizability and clinical applicability [11–14].

In recent years, advances in artificial intelligence (AI) and machine learning (ML) have expanded the methodological options for predictive modeling [15]. The ML has potential value in integrating multi-source clinical data, enabling automated feature selection, and offering greater flexibility in capturing nonlinear patterns [16]. Unbiased extraction of electronic medical records (EMRs) through ML has gained increasing acceptance in clinical research [17]. Despite these advances, clinical adoption of ML-based prediction models remains limited. A key barrier is the “black box” nature of many ML algorithms, where the lack of transparency in decision-making diminishes clinician trust [18]. To address this gap, explainable AI techniques such as SHapley Additive exPlanations (SHAP) have been introduced to elucidate individual feature contributions, thereby enhancing interpretability and supporting clinical decision-making [19]. In this study, we developed and validated an ML model to predict IVIG resistance in children with KD using comprehensive EMR data. This model was evaluated using two external validations and one prospective validation cohort. Model interpretability was enhanced through SHAP-based global and local explanations, and a web-based calculator was constructed to enable real-time, individualized risk estimation, which facilitates early risk stratification.

## Methods

This study was conducted in accordance with the principles outlined in the Declaration of Helsinki. Ethical approval was obtained from the Ethics Committee of the Children’s Hospital of Soochow University (Approval Nos. 2024CS165 and 2023CS244), the Ethics Committee of the Fuzhou University Affiliated Provincial Hospital (Approval No. K2025-02-105), and the Ethics Committee of Yangzhou University Affiliated Hospital (Approval No. 2023-YKL06). Written informed consent was obtained from the legal guardians of all participating children.

### Study cohort and data processing

**Fig. 1:**
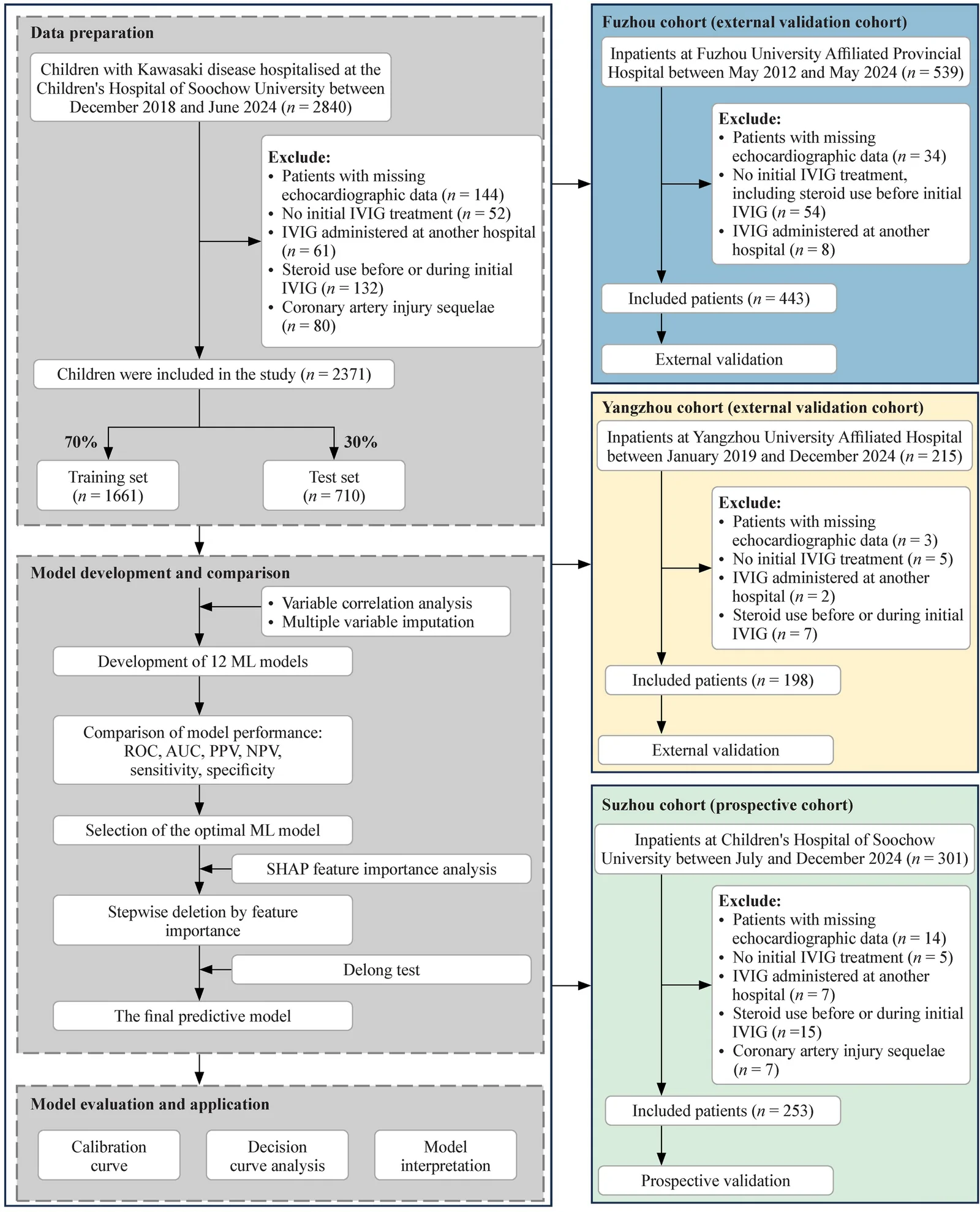
Flowchart of this study. The flowchart illustrates the overall study workflow, including patient selection, data preprocessing, development and comparison of 12 ML models, SHAP-based feature selection, and external/prospective validation of the final predictive model. *IVIG* intravenous immunoglobulin, *ROC* receiver operating characteristic, *AUC* area under the ROC curve, *PPV* positive predictive value, *NPV* negative predictive value, *SHAP* SHapley Additive exPlanations, *ML* machine learning

Based on predefined inclusion and exclusion criteria (Fig. 1 and Supplementary Methods), patients with KD from Suzhou (December 2018–June 2024) were used for model development, with external validation in Fuzhou (May 2012–May 2024) and Yangzhou (January 2019–December 2024), and prospective validation in Suzhou (July 2024–December 2024). KD was diagnosed according to American Heart Association (AHA) criteria [1, 20]. IVIG resistance was defined as fever persisting or recurring within 36 hours after IVIG treatment (2 g/kg), and CALs were defined using the Dallaire *Z* score (*Z* ≥ 2.0) [21]. All predictors were collected prior to treatment.

Clinical and laboratory predictors were extracted from EMRs. Only variables available across all centers and measured using standardized protocols were retained. The Suzhou cohort was randomly split into training (70%) and test (30%) sets. Missing data were handled using multiple imputation by chained equations (MICE) [22], with variables with > 50% missingness excluded and imputation performed separately in the training and test sets to avoid information leakage. Multicollinearity was assessed using Spearman’s correlation analysis. A total of 38 variables were selected for model development (Supplementary Table 1).

### Model development and validation

There are 12 machine learning algorithms were applied for model development. Model training and hyperparameter tuning were performed using repeated 10-fold cross-validation (10 folds, 5 repetitions) within the training set. Model performance was evaluated using area under the receiver operating characteristic (ROC) curve (AUC), sensitivity, specificity, positive predictive value (PPV), and negative predictive value (NPV). The final model was selected based on the highest mean AUC across resampling iterations. Model performance was evaluated in two independent external cohorts and one prospective cohort in terms of discrimination, calibration, and clinical utility.

### Feature selection, interpretation, and clinical utility

The SHAP was used to quantify feature contributions, with low-impact features iteratively removed based on SHAP rankings, recursive feature elimination, and AUC comparisons [23]. Global and local explanations were generated, and interaction effects among predictors were assessed using the interaction method in the “iml” package.

Model calibration was assessed using calibration curves and the Brier score, and clinical utility was evaluated using decision curve analysis (DCA) across clinically relevant threshold probabilities.

An interactive web-based calculator was developed using Shiny to provide individualized IVIG resistance risk prediction, with the classification threshold determined by the Youden index.

### Comparative and sensitivity analyses

Established IVIG resistance scoring systems, including the Kobayashi [7], Egami [6], Sano [8], Formosa [24], and Li [25] scores were implemented using their original definitions without refitting. Performance was evaluated using AUC, sensitivity, and specificity for comparison with the proposed model. To assess the impact of CALs on model performance, additional analyses were conducted by excluding CALs from the candidate predictors, and the entire modeling process was repeated using the same procedures. Model performance was evaluated using AUC, calibration curves, and DCA.

### Reporting and risk of bias assessment

The study was reported in accordance with the transparent reporting of a multivariable prediction model for individual prognosis or diagnosis for AI guidelines (TRIPOD+AI) [26]. Methodological quality, risk of bias, and applicability were assessed using prediction model risk of bias assessment tool for AI (PROBAST+AI) across four domains, with overall judgement based on standard criteria [27]. All statistical analyses were conducted using R (v4.4.2) and SPSS (v25.0; IBM Corp., Armonk, NY, USA). Normality of continuous data was evaluated using the Shapiro-Wilk test. Non-normally distributed variables were expressed as medians with interquartile ranges (IQRs) and compared using the Mann-Whitney *U* test. Categorical variables were reported as frequencies and percentages and compared using the chi-square or Fisher’s exact test, as appropriate. The DeLong test was used to compare AUC values. A two-tailed *P* value < 0.05 was considered statistically significant. Detailed methodology is provided in the Supplementary Materials.

## Results

### Baseline characteristics

As illustrated in Fig. 1, the study workflow involved excluding 469 cases, resulting in a final development cohort of 2371 children with KD from Suzhou. External validation cohorts included 443 patients from Fuzhou and 198 from Yangzhou, while the prospective Suzhou cohort included 253 patients. Among the development cohort, 305 patients (12.9%) were classified as IVIG-resistant. The median age was 26.0 months, and 1444 patients (60.9%) were male. Baseline demographic, clinical, and laboratory characteristics of the cohort are summarized in Table 1. Correlations among variables are presented as a heatmap in Supplementary Fig. 1, where white blood cell (WBC) and neutrophil percentage (NEU%) showed a moderate correlation (*r* = 0.4459), supporting their simultaneous inclusion in the model. The extent of missing data for each variable is detailed in Supplementary Table 1. Compared with IVIG-responsive patients, those who were IVIG-resistant included a significantly higher proportion of males (67.5% vs. 59.9%, *P* = 0.011), as well as an increased incidence of CALs (52.1% vs. 24.7%, *P* < 0.001), rash (83.0% vs. 73.6%, *P* < 0.001), and extremity edema (54.8% vs. 44.3%, *P* = 0.001). In contrast, incomplete Kawasaki disease (iKD) was less common among IVIG-resistant patients than IVIG-responsive patients (18.4% vs. 24.3%, *P* = 0.023).

**Table 1:** Comparison of clinical characteristics and laboratory parameters for study participants

Characteristics	Overall (*n* = 2371)	IVIG-responsive (*n* = 2066)	IVIG-resistant (*n* = 305)	*P* value^*^	Training set (*n* =1661)	Test set (*n* = 710)	*P* value^#^
Male	1444 (60.9)	1238 (59.9)	206 (67.5)	0.011	1005 (60.5)	439 (61.8)	0.545
CALs	670 (28.3)	511 (24.7)	159 (52.1)	< 0.001	476 (28.7)	194 (27.3)	0.509
iKD	558 (23.5)	502 (24.3)	56 (18.4)	0.023	390 (23.5)	168 (23.7)	0.924
Conjunctival injection	2105 (88.8)	1835 (88.8)	270 (88.5)	0.879	1467 (88.3)	638 (89.9)	0.277
Oral mucosal change	2060 (86.9)	1792 (86.7)	268 (87.9)	0.585	1443 (86.9)	617 (86.9)	0.986
Edema of the hands and feet	1082 (45.6)	915 (44.3)	167 (54.8)	0.001	758 (45.6)	324 (45.6)	1.000
Rash	1773 (74.8)	1520 (73.6)	253 (83.0)	< 0.001	1245 (75.0)	528 (74.4)	0.762
Cervical lymphadenopathy	2104 (88.7)	1827 (88.4)	277 (90.8)	0.218	1464 (88.1)	640 (90.1)	0.158
Perianal desquamation	206 (8.7)	179 (8.7)	27 (8.9)	0.913	142 (8.5)	64 (9.0)	0.713
Age at presentation (mon)	26.00 (14.00, 46.00)	27.00 (14.00, 48.00)	24.00 (14.00, 40.00)	0.004	26.00 (14.00, 47.00)	26.00 (14.00, 45.25)	0.906
Time to diagnosis (d)	6.00 (5.00,7.00)	6.00 (5.00, 7.00)	5.00 (4.00, 6.00)	< 0.001	6.00 (5.00, 7.00)	6.00 (4.00, 7.00)	0.055
WBC (×10^9^/L)	12.39 (8.90, 16.38)	12.24 (8.69, 16.10)	13.73 (10.07, 18.98)	< 0.001	12.28 (8.76, 16.46)	12.72 (9.07, 16.36)	0.429
NEU%	60.30 (44.20, 74.60)	59.50 (42.80, 73.90)	67.30 (53.95, 80.30)	< 0.001	60.40 (43.20, 74.85)	60.20 (45.40, 74.33)	0.999
EOS%	2.00 (0.60, 4.10)	2.10 (0.70, 4.20)	1.30 (0.40, 3.10)	< 0.001	2.00 (0.60, 4.10)	2.00 (0.60, 3.90)	0.703
MONO%	6.40 (4.70, 8.40)	6.40 (4.80, 8.40)	5.80 (4.00, 8.35)	0.001	6.40 (4.60, 8.30)	6.30 (4.70, 8.60)	0.529
PLT (×10^9^/L)	365.00 (286.00, 462.00)	369.00 (289.75, 462.00)	330.00 (256.00, 462.00)	0.002	365.00 (284.00, 463.00)	367.00 (290.00, 461.00)	0.617
Hb (g/L)	113.00 (107.00, 120.00)	113.00 (107.00, 120.00)	113.00 (106.00, 120.00)	0.245	114.00 (107.00, 120.00)	113.00 (106.00, 120.00)	0.235
LYMPH%	29.40 (17.80, 43.70)	30.00 (18.70, 44.70)	24.20 (12.80, 34.65)	< 0.001	29.40 (17.65, 43.90)	29.20 (18.10, 42.38)	0.894
CRP (mg/L)	64.11 (37.49, 101.30)	60.83 (34.93, 96.16)	88.96 (58.28, 122.14)	< 0.001	65.11 (37.73, 102.65)	62.31 (36.82, 98.45)	0.409
TG (mmol/L)	1.16 (0.93, 1.48)	1.17 (0.93, 1.48)	1.14 (0.92, 1.49)	0.659	1.17 (0.93, 1.49)	1.16 (0.93, 1.48)	0.774
TC (mmol/L)	3.65 (3.15, 4.15)	3.68 (3.18, 4.18)	3.39 (2.94, 3.93)	< 0.001	3.67 (3.17, 4.16)	3.60 (3.08, 4.09)	0.072
LDH (U/L)	355.50 (297.50, 427.80)	352.80 (296.68, 425.55)	370.20 (301.50, 442.10)	0.056	354.80 (297.80, 425.10)	357.25 (296.33, 432.63)	0.890
GLU (mmol/L)	5.40 (4.80, 6.10)	5.40 (4.80, 6.10)	5.60 (4.90, 6.30)	0.045	5.40 (4.80, 6.10)	5.50 (4.80, 6.20)	0.191
ALB (g/L)	40.00 (37.60, 42.00)	40.00 (37.70, 42.00)	39.20 (36.10, 41.40)	< 0.001	40.00 (37.60, 42.00)	39.80 (37.50, 41.90)	0.330
Ca (mmol/L)	2.18 (1.10, 2.34)	2.18 (1.09, 2.35)	2.19 (1.13, 2.32)	0.570	2.19 (1.10, 2.35)	2.17 (1.09, 2.32)	0.065
CK (U/L)	60.20 (41.30, 88.10)	60.70 (41.60, 89.30)	56.30 (38.55, 81.60)	0.037	59.80 (41.00, 88.75)	60.75 (41.70, 86.98)	0.989
AST (U/L)	33.80 (26.20, 49.90)	33.40 (25.90, 48.73)	36.50 (27.70, 59.45)	0.001	33.80 (25.90, 50.10)	33.70 (26.48, 49.60)	0.802
ALT (U/L)	22.30 (13.50, 66.00)	21.60 (13.30, 58.40)	31.70 (15.05, 119.25)	< 0.001	22.00 (13.70, 67.05)	22.75 (13.28, 64.15)	0.783
CR (µmol/L)	24.60 (20.30, 30.10)	24.55 (20.20, 29.83)	24.90 (20.90, 32.15)	0.015	24.50 (20.30, 30.20)	24.85 (20.38, 29.90)	0.871
GGT (U/L)	20.20 (11.70, 72.50)	19.40 (11.60, 66.23)	28.40 (13.35, 116.50)	< 0.001	19.60 (11.60, 72.60)	21.30 (12.00, 72.45)	0.425
Mg (mmol/L)	0.99 (0.93, 1.06)	0.99 (0.93, 1.06)	0.98 (0.92, 1.06)	0.263	0.99 (0.93, 1.06)	0.98 (0.92, 1.05)	0.136
Na (mmol/L)	135.00 (133.00, 137.00)	135.00 (134.00, 137.00)	135.00 (132.00, 136.50)	< 0.001	135.00 (133.00, 137.00)	135.00 (133.00, 137.00)	0.537
K (mmol/L)	3.90 (3.60, 4.30)	3.90 (3.60, 4.20)	3.90 (3.55, 4.30)	0.938	3.90 (3.60, 4.30)	3.90 (3.60, 4.23)	0.850
Cl (mmol/L)	103.00 (102.00, 105.00)	103.00 (102.00, 105.00)	103.00 (101.00, 105.00)	0.420	103.00 (102.00, 105.00)	103.00 (101.00, 105.00)	0.779
ALP (U/L)	175.00 (147.00, 222.00)	174.00 (145.00, 219.00)	190.00 (156.00, 237.50)	0.001	175.00 (147.00, 219.00)	181.00 (147.00, 225.00)	0.358
BUN (mmol/L)	3.04 (2.39, 3.75)	3.00 (2.37, 3.70)	3.26 (2.46, 4.22)	< 0.001	3.04 (2.39, 3.74)	3.04 (2.39, 3.78)	0.899
CKMB (ng/mL)	1.20 (0.80, 1.90)	1.20 (0.78, 1.90)	1.20 (0.80, 1.90)	0.829	1.20 (0.78, 1.90)	1.20 (0.80, 1.90)	0.739
UWBC (/µL)	7.40 (2.30, 23.30)	7.00 (2.30, 21.70)	11.00 (3.00, 37.50)	0.001	7.70 (2.30, 24.00)	6.70 (2.38, 21.55)	0.498

Median (interquartile range, IQR) is used for continuous variables, and number (%) for categorical variables. *P* < 0.05 is considered as statistically significant. ^*^Comparisons between the IVIG-responsive and IVIG-resistant groups. ^#^Comparisons between the training and test sets. Categorical variables were compared using the chi-square test or Fisher’s exact test, and continuous variables using the Mann-Whitney *U* test. *IVIG *intravenous immunoglobulin, *CALs *coronary artery lesions, *iKD *incomplete Kawasaki disease, *WBC *white blood cell, *NEU%* neutrophil percentage, *EOS%* eosinophil percentage, *MONO% *monocyte percentage, *PLT *platelet, *Hb *hemoglobin, *LYMPH%* lymphocyte percentage, *CRP *C-reactive protein, *TG *triglyceride, *TC *total cholesterol, *LDH* lactate dehydrogenase, *GLU* glucose, *ALB* albumin, *Ca* calcium, *CK* creatine kinase, *AST* aspartate aminotransferase, *ALT* alanine aminotransferase, *CR* creatinine, *GGT* γ-glutamyl transpeptidase, *Mg* magnesium, *Na* sodium, *K* potassium, *Cl *chloride, *ALP *alkaline phosphatase, *BUN *blood urea nitrogen, *CKMB *creatine kinase-MB, *UWBC *urinary white blood cell

Several laboratory parameters were elevated in the IVIG-resistant group, including WBC, NEU%, C-reactive protein (CRP), urinary WBC (UWBC), alanine aminotransferase (ALT), aspartate aminotransferase (AST), alkaline phosphatase (ALP), γ-glutamyl transpeptidase (GGT), creatinine (CR), blood urea nitrogen (BUN), and glucose (GLU); all with *P* < 0.05. Baseline clinical and laboratory characteristics did not differ significantly between the training set (*n* = 1661) and the test set (*n* = 710), supporting internal consistency (*P* > 0.05).

### Model development and feature selection

**Fig. 2:**
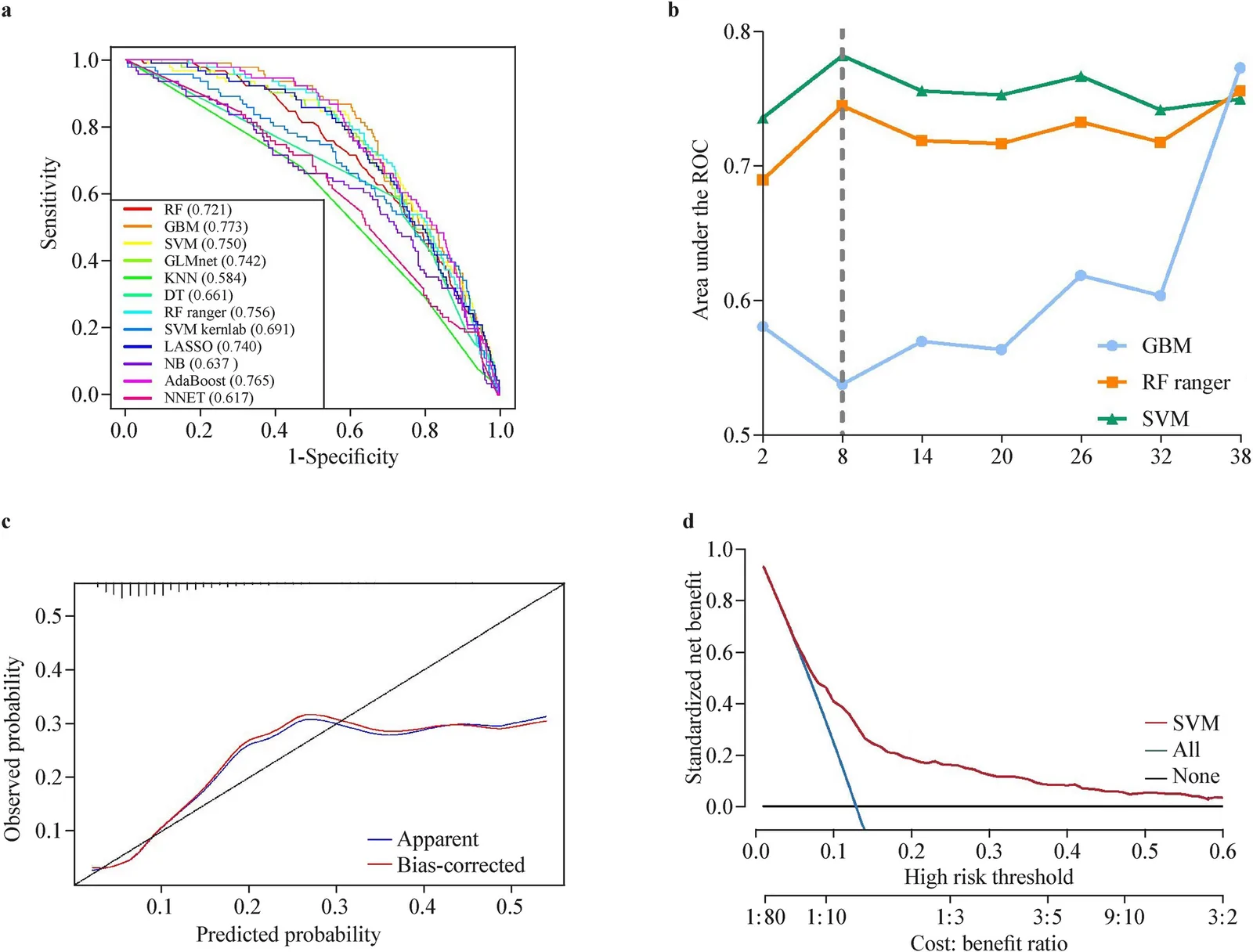
Development and internal validation of machine learning models for predicting IVIG resistance. **a** ROC curves of all evaluated models; **b** Changes in AUC during stepwise feature elimination, with the vertical dashed line indicating the final eight-feature model; **c** Calibration curves for internal validation of the final SVM model, where the black diagonal line represents ideal calibration and the blue and red lines represent the apparent and bias-corrected curves, respectively; **d** DCA for internal validation, where the red, blue, and black lines represent the model, treat-all, and treat-none strategies, respectively. *IVIGR* intravenous immunoglobulin resistance, *ROC* receiver operating characteristic, *AUC* area under the ROC curve, *RF* random forest, *GBM* gradient boosting machine, *GLMnet* generalized linear model with elastic net regularization, *SVM* support vector machine, *LASSO* least absolute shrinkage and selection operator, *DT* decision tree, *KNN* K-nearest neighbor, *NB* naïve Bayes, *Adaboost* adaptive boosting, *NNET* neural network, *RF* ranger, RF with ranger algorithm, *SVM kernlab*, SVM with kernlab algorithm, *DCA* decision curve analysis

The AUC for each model is presented in Fig. 2a, and corresponding performance metrics are summarized in Supplementary Table 2. The five top-performing models were gradient boosting machine (GBM) (AUC = 0.773), adaptive boosting (AdaBoost) (AUC = 0.765), random forest with ranger algorithm (RF ranger) (AUC = 0.756), support vector machine (SVM) (AUC = 0.750), and generalized linear model with elastic net (GLMnet) (AUC = 0.742). Based on both AUC and balanced sensitivity/specificity, GBM, SVM, and RF ranger were selected for further refinement.

The ten most influential predictors for each model, ranked by SHAP values, are displayed in Supplementary Fig. 2. Common top features across all three models included CALs, CRP, age at presentation, NEU%, and WBC. Stepwise elimination of less influential variables was performed, with the corresponding changes in AUC, sensitivity, and specificity presented in Fig. 2b, Supplementary Fig. 3, and Supplementary Table 3. Among the three algorithms, SVM maintained the highest AUC throughout feature reduction, leading to its selection as the final model. The optimal model using eight features outperformed both the 14-feature model in AUC (0.782 vs. 0.756; DeLong test, *P* = 0.025) and a minimal two-feature model (0.782 vs. 0.736; *P* = 0.009). The final SVM model included CALs, CRP, age at presentation, NEU%, WBC, total cholesterol (TC), platelets (PLT), and serum albumin (ALB).

**Fig. 3:**
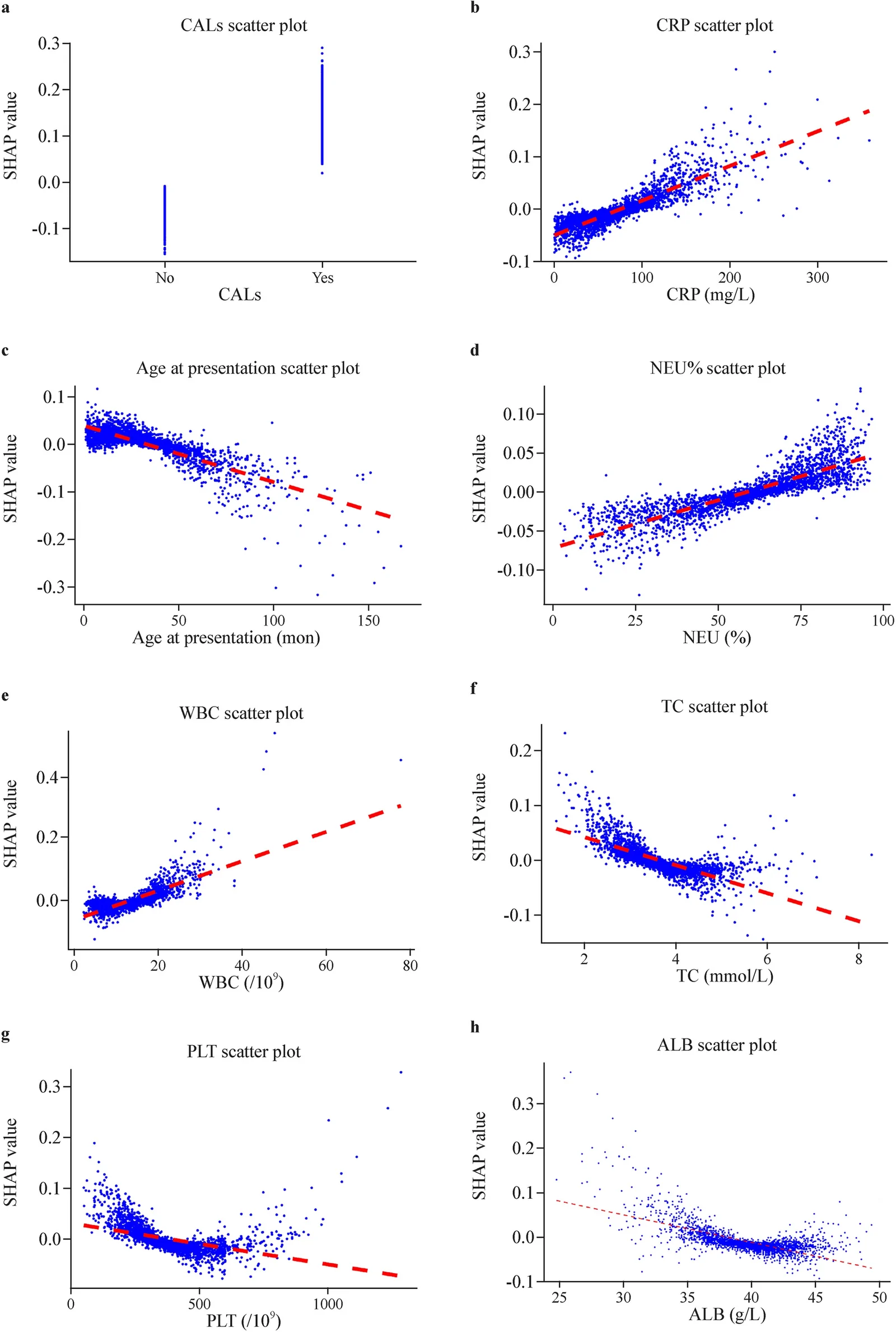
SHAP-based feature importance visualization. Scatterplots of SHAP values vs. feature values for the eight key predictors in the final SVM model. The y-axis represents SHAP values, and the x-axis represents the actual feature values. A SHAP value greater than 0 indicates a positive contribution to IVIG resistance prediction. The red dashed lines represent fitted trend lines between feature values and SHAP values. **a** CALs scatter plot; **b** CRP scatter plot; **c** Age at presentation scatter plot; **d** NEU% scatter plot; **e** WBC scatter plot; **f** TC scatter plot; **g** PLT scatter plot; **h** ALB scatter plot. *IVIG* intravenous immunoglobulin, *SHAP* SHapley Additive exPlanations, *CALs* coronary artery lesions, *CRP* C-reactive protein, *NEU%* neutrophil percentage, *WBC* white blood cell, *TC* total cholesterol, *PLT* platelet, *ALB* serum albumin

In the internal cohort, the final SVM model demonstrated strong calibration, with predicted probabilities closely matching observed probabilities (Fig. 2c), and provided greater net clinical benefit than the “treat-all” or “treat-none” strategies across a threshold range of 0.04–0.60 (Fig. 2d). SHAP scatter plots (Fig. 3a–h) illustrate the relationship between individual features and IVIG resistance risk. CRP, NEU%, WBC, and CALs were positively associated with increased risk, while older age, higher TC, PLT, and ALB levels were protective. For example, WBC greater than 21.15 ×10⁹/L and the presence of CALs yielded positive SHAP values, indicating increased risk. In contrast, patients aged older than 65 months showed predominantly negative SHAP values.

Local SHAP explanations for four representative patients are provided in Supplementary Fig. 4. Risk factors (red bars) and protective factors (blue bars) varied by patient. For instance, Patient 1 was IVIG-responsive due to protective contributions from the absence of CALs, lower WBC, and higher TC. Patients 2 and 4 were IVIG-resistant primarily due to the presence of CALs. Global interaction-strength analysis of the final SVM model showed that ALB had the highest overall interaction strength, followed by CRP, PLT, and TC (Supplementary Fig. 5). This pattern suggests that the contributions of these predictors to model output were partly dependent on their interactions with other variables. This indicated that the final model captured not only individual predictor effects but also non-additive relationships among clinical and laboratory features associated with IVIG resistance.

### Web-based calculator and validation performance

**Fig. 4:**
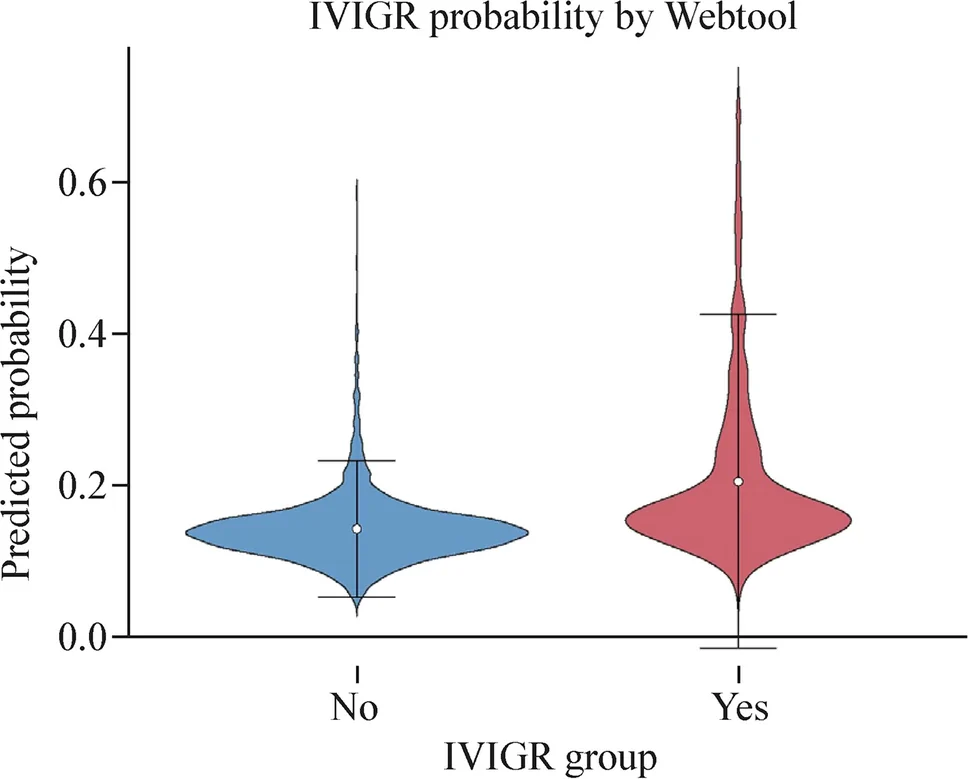
Predicted probability distribution of IVIG resistance by the final SVM model. Violin plots show the distribution of predicted probabilities in IVIG-responsive and IVIG-resistant groups. The width of each violin reflects patient density at each predicted probability level. White dots indicate the mean predicted probability, and black error bars represent the mean ± 2 standard deviations. The higher and broader distribution with a longer upper tail in the IVIG-resistant group indicates generally higher predicted risks, whereas the overlap between groups supports use of the tool for risk stratification rather than as an absolute cutoff. *IVIGR* intravenous immunoglobulin resistance

To enhance clinical utility, an interactive web-based calculator was developed and deployed via Shiny (https://ymswhuang.shinyapps.io/data/). Users can input patient-specific values for the eight features to obtain an individualized risk estimate of IVIG resistance, visually represented by color-coded outputs. The optimal threshold for risk stratification was set at 14.9%, as determined by the Youden Index, yielding a sensitivity of 0.648 and specificity of 0.606. The violin plot showed that IVIG-resistant patients had generally higher predicted probabilities than IVIG-responsive patients, as reflected by the upward shift and longer upper tail of the distribution (Fig. 4).

**Fig. 5:**
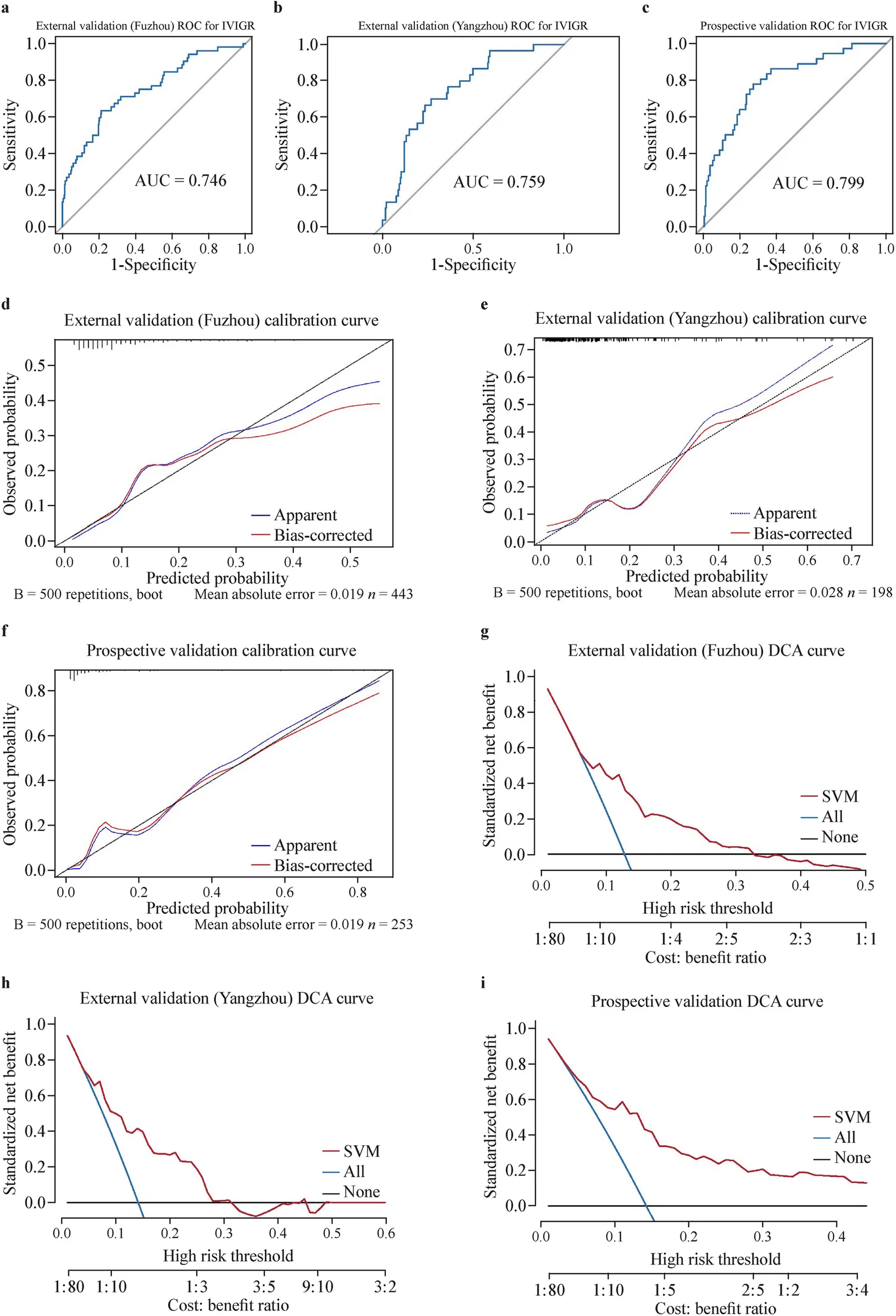
External and prospective validation of the final SVM model. **a** ROC curves for external validation using the Fuzhou cohort; **b** External validation using the Yangzhou cohort; **c** Prospective validation using the Suzhou cohort. In panels **a**–**c**, the blue solid lines represent the ROC curves of the final SVM model across different prediction thresholds, and the gray diagonal lines represent the no-discrimination reference line corresponding to random classification (AUC = 0.5). Curves closer to the upper-left corner indicate better discrimination. **d** Calibration curves for external validation (Fuzhou), **e** External validation (Yangzhou), **f** Prospective validation (Suzhou). In **d**–**f**, the diagonal black lines represent ideal calibration, and the blue and red lines represent the apparent and bias-corrected curves, respectively. **g** DCA for external validation (Fuzhou); **h** External validation (Yangzhou); **i** Prospective validation (Suzhou). In **g**–**i**, the red, blue, and black lines represent the model, treat-all, and treat-none strategies, respectively. *IVIGR* intravenous immunoglobulin resistance, *ROC* receiver operating characteristic, *AUC* area under the ROC curve, *DCA* decision curve analysis, *SVM* support vector machine

The generalizability of the final SVM model was evaluated using external and prospective datasets. In the external cohorts, the model achieved an AUC of 0.746 in Fuzhou (Fig. 5a) and 0.759 in Yangzhou (Fig. 5b), while in the prospective Suzhou cohort, the AUC reached 0.799 (Fig. 5c), indicating moderate predictive performance across centers. Calibration analysis showed good agreement between predicted and observed probabilities (Fig. 5d–f), with Brier scores of 0.099 in Fuzhou, 0.097 in Yangzhou and 0.068 in the prospective Suzhou cohort. DCA demonstrated greater net clinical benefit than the ‘treat-all’ or ‘treat-none’ strategies across threshold probability ranges of 0.05–0.32 in Fuzhou, 0.05–0.31 in Yangzhou, and 0.05–0.40 in the prospective cohort (Fig. 5g–i). Comparative analyses of the eight key features between IVIG-responsive and -resistant patients are shown in Supplementary Table 4.

### Subgroup and comparative analyses

Among patients aged 0–5 months, only NEU% and TC differed significantly between groups. Among patients aged 6–11 months, CALs, CRP, and NEU% were significantly different. Among children aged 1–2 years, multiple variables, including CALs, CRP, NEU%, WBC, TC, and ALB, showed significant differences. In patients aged 3–4 years, seven features (CALs, CRP, NEU%, WBC, TC, ALB, and PLT) were significantly associated with IVIG resistance. In contrast, in patients aged older than five years, only CALs and TC remained statistically significant (Supplementary Table 5).

For comparison, a conventional logistic regression (LR) model was constructed using the same eight predictors. The LR model achieved an AUC of 0.732 [95% confidence interval (95% CI) 0.680–0.784], with a sensitivity of 0.780, specificity of 0.612, PPV of 0.228, and NPV of 0.950. The SVM model achieved an AUC of 0.782 (95% CI 0.739–0.824). Although the AUC of the SVM model was slightly higher than that of the LR model, there was overlap between their confidence intervals, and the difference between the two models was not statistically significant according to the DeLong test (*P* = 0.145).

In our cohort, we compared the final model with established IVIG resistance scoring systems, including Kobayashi, Egami, Sano, Formosa, and Li. As shown in Supplementary Table 6, the AUCs of the Kobayashi, Egami, Sano, Formosa, and Li scores were 0.655, 0.653, 0.672, 0.619, and 0.674, respectively, all lower than that of our SVM model (AUC = 0.782), which also demonstrated balanced performance in terms of sensitivity (0.735) and specificity (0.721).

### Model performance after excluding coronary artery lesions

To provide early and timely risk assessment of IVIG resistance for patients who cannot undergo prompt echocardiography, we further conducted model reconstruction excluding CALs from the candidate predictors. Among the evaluated algorithms, the performances of SVM with the kernlab algorithm (SVM kernlab), least absolute shrinkage and selection operator (LASSO), and GLMnet were comparable in AUC (0.739, 0.740, and 0.739, respectively; Supplementary Fig. 6a, Supplementary Table 7). In the stepwise variable reduction process of the SVM kernlab model (Supplementary Fig. 6b, Supplementary Table 8), the five-variable model performed better than the two-variable model (0.762 vs. 0.704; DeLong test, *P* < 0.001) and also outperformed the eight-variable model (0.762 vs. 0.719; DeLong test, *P* = 0.012), leading to the five-variable model being identified as the optimal solution. In comparison with traditional regression models, LASSO retained 12 variables and GLMnet retained 18 variables (Supplementary Fig. 7).

In an overall comparison, the SVM model including CALs (AUC = 0.750) and the SVM kernlab model excluding CALs (AUC = 0.739) showed no statistically significant difference (DeLong test, *P* = 0.393). We also evaluated the model excluding CALs following the same procedures as described previously. Supplementary Fig. 8–9 illustrate the global and local interpretability of this model. In the validation phase, the SVM kernlab model demonstrated stable discriminative ability in both the external and prospective cohorts (Supplementary Fig. 10). Calibration curves indicated good agreement between predicted and observed outcomes across the internal, external, and prospective cohorts (Supplementary Fig. 11a–c). Furthermore, DCA showed that the SVM kernlab model without CALs continued to provide consistent net clinical benefit across clinically relevant thresholds (Supplementary Fig. 11d–f).

### Reporting and risk of bias assessment

This study was conducted in accordance with TRIPOD+AI guidelines (Supplementary Table 9). The PROBAST+AI assessment indicated a high risk of bias in the participants domain due to the retrospective design, and in the predictors’ domain because blinding of predictor assessment to outcome status could not be ensured. The analysis domain was also judged as high risk, as sample size considerations were not formally assessed. In contrast, the outcome domain was rated as low risk. Applicability across participants, predictors, and outcome domains were considered to be of low concern (Supplementary Table 10).

## Discussion

In this study, we developed and evaluated 12 ML models for predicting IVIG resistance in a cohort of 2371 patients with KD. The observed IVIG resistance rate of 12.9% aligns with previous reports from Chinese cohorts [28, 29]. Traditional risk scoring systems, including the Kobayashi [7], Egami [6], and Sano [8] scores, showed limited predictive performance in our population, likely due to demographic variability. By leveraging SHAP for feature selection, we identified eight key predictors (CALs, CRP, age at presentation, NEU%, WBC, TC, PLT, and ALB) and constructed a final SVM model. This SVM-based model achieved moderate predictive performance, with an AUC of 0.782 in internal testing, 0.746 in external validation from Fuzhou, 0.759 in external validation from Yangzhou, and 0.799 in prospective validation. These results support the generalizability of the model and suggest its potential for real-world clinical implementation. As a supervised ML method, SVM is particularly effective in handling high-dimensional, nonlinear classification problems by constructing optimal separating hyperplanes [30, 31].

### Key predictors of intravenous immunoglobulin resistance

#### Role of coronary artery lesions in prediction

Amongst all predictive features, CALs consistently ranked as the most influential variable across models and in local case analyses, underscoring their central role in risk stratification. These findings support existing evidence that excessive immune activation in resistant patients contributes to vascular injury [32]. Dominguez et al*.* found that most coronary abnormalities were present before IVIG, with 81% detected on initial echocardiogram, indicating a need for more aggressive initial therapy [33]. Previous studies have incorporated CALs into IVIG resistance prediction models [34–37]. The relatively high prevalence of CALs (28.3%) in our cohort may be partly explained by the use of pre-treatment echocardiographic assessments, as well as the inclusion of patients from a tertiary referral center, where more severe disease or delayed diagnosis is common. Of note, differences in outcome prevalence and case mix across populations may affect model calibration and generalizability [38]. However, as early risk assessment is often required before echocardiography and CALs may partly reflect disease severity, we further evaluated a model excluding CALs. Model discrimination remained stable (with CALs: 0.750 vs. without CALs: 0.739, *P* = 0.393), and a CALs-free model was developed to enhance applicability in settings where echocardiography is not immediately available.

#### Predictive role of age and its heterogeneity

Our analysis confirmed younger age as an independent risk factor for IVIG resistance, consistent with previous prediction models such as Egami and Kobayashi, which include age < 6 or ≤ 12 months as key criteria [6, 7]. Lim et al. reported a resistance rate as high as 31.5% among infants under 12 months [39] and a recent meta-analysis further validated age as a significant predictive factor [40]. This association is likely due to immune system immaturity in infants, which may exacerbate inflammatory responses and reduce the efficacy of IVIG. Younger children often exhibit atypical or incomplete KD presentations, potentially delaying diagnosis and treatment [28, 39].

The distribution and inflammatory responsiveness of biomarkers may vary substantially with age, and such heterogeneity may influence their associations with clinical outcomes. Previous studies have also shown that KD presents with distinct clinical features across different age groups [41, 42]. However, it is noteworthy that modeling age as a single variable may not fully capture potential effect modification between age and individual biomarkers. Prior evidence suggests that the associations between some biomarkers, such as platelet count, and IVIG resistance or coronary artery outcomes may be age-dependent [43]. Consistent with this, our additional analyses revealed clear age-related heterogeneity in biomarker associations. Future studies may benefit from age-stratified or interaction-based modeling approaches to better characterize such heterogeneity.

#### Role of laboratory markers in prediction

Laboratory findings revealed that elevated CRP, NEU%, and WBC levels, along with reduced PLT, ALB, and TC, were significantly associated with IVIG resistance. Indeed, these markers have been previously reported as reliable predictors [1, 44–47]. High NEU% contributes to vascular injury by releasing reactive oxygen species, proteases, and proinflammatory cytokines [36, 48, 49]. This is supported by autopsy studies showing dense neutrophilic infiltration in coronary lesions [50, 51]. Similarly, elevated WBC has been repeatedly linked to treatment resistance [29]. Low PLT, as observed in the present study, have also been associated with IVIG resistance [52]. Thrombocytopenia may be related to the persistent consumption of platelets at sites of coronary artery injury, where platelets adhere to exposed collagen and become excessively activated, subsequently releasing vascular endothelial growth factor (VEGF) and matrix metalloproteinases (MMPs), which further aggravate vascular injury and remodeling [2, 53]. Hypoalbuminemia may reflect increased vascular permeability and impaired hepatic function due to systemic inflammation [54]. Although the mechanistic link between low TC and IVIG resistance remains unclear, prior studies have observed significant lipid abnormalities in resistant patients, including reduced levels of TC, high-density lipoprotein cholesterol (HDL-C), low-density lipoprotein cholesterol (LDL-C), and apolipoprotein A, alongside elevated triglycerides [55].

### Dataset independence and temporal heterogeneity

The external validation cohorts were derived from independent institutions that were not involved in model development, ensuring institutional independence. Clinical management across centers followed AHA guidelines [1, 20], and standardized procedures were used for data collection and processing, with no overlap in patient records. The development cohort in Suzhou was collected between December 2018 and June 2024, whereas the external validation cohorts included patients from Fuzhou collected between May 2012 and May 2024 and Yangzhou collected between January 2019 and December 2024. Such temporal differences may introduce heterogeneity, as changes in patient characteristics, clinical practice, and outcome incidence over time could influence model performance. In this context, model discrimination may remain relatively stable, whereas calibration is more susceptible to variation and may exhibit systematic deviations between predicted and observed risks, a phenomenon known as calibration drift [56].

### Clinical applications and interpretability

To ensure transparency and reliability of prediction models, the updated TRIPOD+AI reporting guideline emphasizes the need to comprehensively report both calibration performance and clinical utility [26]. Shah et al. further highlighted that without systematic assessment of calibration and clinical benefit, prediction models may remain academic exercises rather than clinically applicable tools [57]. In our study, calibration curves showed good agreement between predicted and observed outcomes, and DCA indicated that the SVM model achieved greater net clinical benefit than the default strategies of treating all or treating none, supporting its clinical validity. Moreover, SHAP enhances the transparency of machine learning models by providing interpretable, individualized explanations, a critical factor for clinical adoption. To facilitate clinical use, we developed a web-based risk calculator that enables real-time, patient-specific prediction. The optimal threshold (14.9%) was determined using the Youden index to balance sensitivity and specificity. However, as this approach is primarily based on statistical optimization, it should be interpreted with caution [58]. Importantly, reliance on a single fixed threshold may not be suitable for routine clinical practice. Of note, this threshold lies within the range of clinically relevant probabilities identified by DCA (0.04–0.60 in internal validation, 0.05–0.32 in Fuzhou, 0.05–0.31 in Yangzhou, and 0.05–0.40 in the prospective cohort), supporting clinical acceptability. In practice, this threshold may serve as a reference for early risk stratification rather than a strict treatment cutoff, and clinical decisions should be guided by comprehensive clinical judgment [59].

### Methodological considerations

This study aimed to balance predictive performance, interpretability, and clinical applicability by systematically evaluating multiple representative algorithms and selecting a parsimonious model suitable for clinical use. At the same time, existing evidence suggests that there is currently no universally accepted “gold standard” method for clinical prediction modeling, and model performance largely depends on dataset characteristics (such as sample size, class imbalance, nonlinearity, and the number of candidate predictors) as well as data quality. Therefore, model selection should be guided by the specific clinical question and data structure [60]. In this study, the CIs of the SVM and logistic regression models overlapped, and the difference in AUC between the two models was not statistically significant, suggesting comparable discriminatory performance. Previous studies have also shown that LR can perform as well as ML models in predicting major chronic disease risk [61]. In particular, in settings with moderate sample sizes, a limited number of events, and structured clinical data, conventional approaches (e.g., logistic regression) remain practical and appropriate, while for tabular data, tree-based models may perform comparably to, or even outperform, more complex deep learning approaches [16, 61, 62].

Compared with previous studies, our work was based on a relatively large multicenter cohort and further supported by external validation in two independent cohorts, as well as prospective validation, which may enhance the generalizability of the model. In addition, we conducted a comprehensive performance evaluation in accordance with current methodological recommendations, including discrimination, calibration, decision curve analysis, and the Brier score, and incorporated SHAP-based and interaction analyses to improve interpretability. Furthermore, adherence to TRIPOD+AI and PROBAST+AI guidelines enhanced the transparency and methodological rigor of the study.

Future studies may further integrate multi-omics (e.g., genomics and proteomics) and imaging data to explore the potential application of more advanced ML approaches and potentially improve model performance. We also acknowledge that ensemble methods and emerging tabular foundation models (e.g., TabPFN) hold potential value and warrant further investigation in future research [63]. TabPFN is specifically designed for tabular data and optimized for structured inputs, with the capability to handle complex feature interactions and potentially improve predictive performance [63]. In addition, exploring alternative clinically relevant outcomes, such as hospital readmission and recurrent KD, may further extend the applicability of prediction models.

This study still has several limitations. First, although it included both retrospective and prospective data, the retrospective design of the primary cohort inherently increases the risk of bias. Broader, multicenter validation is needed to confirm generalizability across populations. Second, while standardized diagnostic criteria were employed, the diagnosis of KD may still involve some degree of clinical subjectivity. Third, although age was included as a predictor, modeling it as a single variable may not fully capture potential age-related heterogeneity or effect modification between age and individual biomarkers. Fourth, the analysis relied on structured data from EMRs, and only summary echocardiographic findings were available for review, not raw images. The absence of genetic, multi-omics, or imaging-derived biomarkers may have limited the predictive scope of the model. Furthermore, interobserver variability in echocardiographic interpretation could have influenced outcome assessment. Finally, prospective trials with propensity score matching are needed to determine whether model-guided decision-making improves clinical outcomes in practice.

In conclusion, this study developed and validated an SVM-based machine learning model for predicting IVIG resistance in patients with Kawasaki disease. The model incorporated eight key features, with CRP, NEU%, WBC, and CALs positively associated with resistance, and older age, higher TC, PLT, and ALB levels serving as protective factors. The model demonstrated acceptable discrimination, good calibration, and favorable clinical utility as assessed by DCA. Future studies should adhere more strictly to TRIPOD+AI and PROBAST+AI guidelines and incorporate multi-omics data to further improve model performance and clinical applicability.

## Supplementary Information

Below is the link to the electronic supplementary material.

Supplementary file1 (DOCX 3190 KB)

## Data Availability

The datasets generated and analyzed during the current study are publicly available on the Figshare repository at the following links: https://doi.org/10.6084/m9.figshare.28444157.v1; https://doi.org/10.6084/m9.figshare.28516721.
