# Supplementary material for: Machine learning model for intravenous immunoglobulin resistance in Kawasaki disease: model development and validation study
Source: World J Pediatr. 2026 Sep 1;22(8):939–54. doi: 10.1007/s12519-026-01075-w (PMC13615047; doi:10.1007/s12519-026-01075-w)
Supplement: Supplementary file 1 [file 12519_2026_1075_MOESM1_ESM.docx]

**Supplementary information**

**Supplementary methods**

***Diagnostic and exclusion criteria***

Complete Kawasaki disease (cKD) was defined as fever plus at least four of the five principal clinical features, including conjunctival injection, oral mucosal changes, rash, extremity changes, and cervical lymphadenopathy, according to American Heart Association (AHA) criteria [1, 2]. In line with guideline recommendations, when ≥ four principal clinical features are present, the diagnosis can be established with four days of fever; in selected cases, experienced clinicians may establish the diagnosis earlier, at three days of fever. Incomplete KD (iKD) was defined as fever with 2-3 principal clinical features accompanied by elevated inflammatory markers, including C-reactive protein (CRP ≥ 30 mg/L) or erythrocyte sedimentation rate (ESR ≥ 40 mm/h).

Transthoracic echocardiography was performed at baseline, prior to intravenous immunoglobulin (IVIG) treatment, using standardized equipment (PHILIPS and GE). Examinations were conducted by two experienced pediatric cardiologists (each with experience over five years) and independently confirmed by a third senior cardiologist to ensure consistency.

Patients were excluded if they met any of the following criteria: (1) lack of echocardiography data; (2) no IVIG treatment during initial therapy; (3) receipt of IVIG at another institution prior to admission; (4) administration of steroids prior to or concurrent with initial IVIG; or (5) being in the coronary artery sequelae phase due to prior KD history and persistent coronary artery dilation [3].

***Data collection and missing data handling***

Data were categorized into four domains: demographic information, clinical features, laboratory parameters, and echocardiographic findings. All centers followed standardized procedures for data collection and processing, and no overlap in patient records was observed between cohorts.

Detailed missing rates of all candidate predictors, quantified separately in the training and test sets, are provided in Supplementary Table 1. Multiple imputation by chained equations (MICE) was performed with 20 imputations and 50 iterations per chain. Continuous variables were imputed using predictive mean matching (pmm), binary variables with logistic regression, and categorical variables with polytomous regression. The outcome variable (IVIG resistance) was not imputed but included as a predictor. Continuous variables were retained in their original form, while categorical variables were ordinally encoded.

Two researchers independently and randomly verified 20% of the extracted data for accuracy. Clinically similar or redundant features were further reviewed by two clinicians (JZ and HH), with disagreements resolved through expert adjudication (HL).

***Machine learning algorithms***

Twelve algorithms were applied for model development, including random forest (RF) [4], support vector machine (SVM) [5], gradient boosting machine (GBM) [6], generalized linear model with elastic net regularization (GLMnet) [7], decision tree (DT) [8], K-nearest neighbor (KNN) [9], RF with ranger algorithm (RF ranger) [10], SVM with kernlab algorithm (SVM kernlab) [11], least absolute shrinkage and selection operator (LASSO) [12], adaptive boosting (AdaBoost) [13], naïve Bayes (NB) [14], and neural network (NNET) [15].

***Detailed implementation of established IVIG resistance scoring systems***

For the Kobayashi score, points were assigned according to predefined thresholds for serum sodium, illness days at initial treatment, aspartate aminotransferase, neutrophil percentage, C-reactive protein (CRP), age, and platelet count (PLT). For the Egami score, variables included alanine aminotransferase, illness days at initial treatment, CRP, age, and PLT. The Sano, Formosa, and Li scores were similarly calculated based on their original criteria.

To ensure consistency, variable units were harmonized with the original definitions. Specifically, C-reactive protein values recorded in mg/L were converted to equivalent mg/dL thresholds where required, and platelet counts recorded as ×10^9^/L were adjusted accordingly. Age at presentation was directly mapped to the original scoring criteria.

***PROBAST+AI assessment details***

The methodological quality and risk of bias of the prediction model were assessed using the prediction model risk of bias assessment tool for AI (PROBAST+AI) tool across four domains (participants, predictors, outcome, and analysis), with separate evaluations performed for the model development and evaluation phases using predefined signalling questions. Each item was rated as Yes, Probably Yes, Probably No, No, No Information, or Not Applicable. A domain was judged as low risk if all items were rated Yes or Probably Yes, high risk if any item was rated Probably No or No, and otherwise as unclear. Applicability was evaluated based on the participants, predictors, and outcome domains using the same criteria. The overall judgement was defined as low risk when all domains were rated as low risk, high risk when any domain was rated as high risk, and unclear otherwise.

**Supplementary tables**

**Supplementary Table 1 Missing data for each variable in the training and test sets.**

| Variable | Missing data, *n* (Training set) | Percentage of missing data, % | Missing data, *n* (Test set) | Percentage of missing data, % |
| --- | --- | --- | --- | --- |
| Gender | 1 | 0.06 | 0 | 0.00 |
| CALs | 0 | 0.00 | 0 | 0.00 |
| iKD | 0 | 0.00 | 0 | 0.00 |
| Conjunctival injection | 1 | 0.06 | 0 | 0.00 |
| Oral mucosal change | 1 | 0.06 | 0 | 0.00 |
| Edema of the hands and feet | 1 | 0.06 | 0 | 0.00 |
| Rash | 1 | 0.06 | 0 | 0.00 |
| Cervical lymphadenopathy | 1 | 0.06 | 1 | 0.14 |
| Perianal desquamation | 2 | 0.12 | 1 | 0.14 |
| Age at presentation | 1 | 0.06 | 0 | 0.00 |
| Time to diagnosis | 3 | 0.18 | 0 | 0.00 |
| WBC | 2 | 0.12 | 0 | 0.00 |
| NEU% | 2 | 0.12 | 0 | 0.00 |
| EOS% | 2 | 0.12 | 0 | 0.00 |
| MONO% | 2 | 0.12 | 0 | 0.00 |
| PLT | 2 | 0.12 | 0 | 0.00 |
| Hb | 0 | 0.00 | 0 | 0.00 |
| LYMPH% | 2 | 0.12 | 0 | 0.00 |
| CRP | 4 | 0.24 | 5 | 0.70 |
| TG | 9 | 0.54 | 8 | 1.13 |
| TC | 9 | 0.54 | 8 | 1.13 |
| LDH | 9 | 0.54 | 8 | 1.13 |
| GLU | 38 | 2.29 | 14 | 1.97 |
| ALB | 9 | 0.54 | 8 | 1.13 |
| Ca | 9 | 0.54 | 6 | 0.85 |
| CK | 9 | 0.54 | 8 | 1.13 |
| AST | 9 | 0.54 | 8 | 1.13 |
| ALT | 9 | 0.54 | 8 | 1.13 |
| CR | 8 | 0.48 | 8 | 1.13 |
| GGT | 9 | 0.54 | 8 | 1.13 |
| Mg | 222 | 13.37 | 99 | 13.94 |
| Na | 38 | 2.29 | 14 | 1.97 |
| K | 38 | 2.29 | 14 | 1.97 |
| Cl | 38 | 2.29 | 14 | 1.97 |
| ALP | 9 | 0.54 | 8 | 1.13 |
| BUN | 8 | 0.48 | 8 | 1.13 |
| CKMB | 301 | 18.12 | 128 | 18.03 |
| UWBC | 0 | 0.00 | 0 | 0.00 |
| IVIGR | 0 | 0.00 | 0 | 0.00 |

*IVIGR,* intravenous immunoglobulin resistance; *CALs*, coronary artery lesions; *iKD*, incomplete Kawasaki disease; *WBC*, white blood cell; *NEU%*, neutrophil percentage; *EOS%*, eosinophil percentage; *MONO%*, monocyte percentage; *PLT*, platelet; *Hb*, hemoglobin; *LYMPH%*, lymphocyte percentage; *CRP*, C-reactive protein; *TG*, triglyceride; *TC*, total cholesterol; *LDH*, lactate dehydrogenase; *GLU*, glucose; *ALB*, albumin; *Ca*, calcium; *CK*, creatine kinase; *AST*, aspartate aminotransferase; *ALT*, alanine aminotransferase; *CR*, creatinine; *GGT*, γ-glutamyl transpeptidase; *Mg*, magnesium; *Na*, sodium; *K*, potassium; *Cl*, chloride; *ALP*, alkaline phosphatase; *BUN*, blood urea nitrogen; *CKMB*, creatine kinase-MB; *UWBC*, urinary white blood cell.

**Supplementary Table 2 Performance of ML models for IVIG resistance in KD.**

| Model | AUC | Sensitivity | Specificity | PPV | NPV |
| --- | --- | --- | --- | --- | --- |
| RF | 0.721 | 0.989 | 0.022 | 0.873 | 0.222 |
| GBM | 0.773 | 0.716 | 0.571 | 0.919 | 0.228 |
| SVM | 0.750 | 0.714 | 0.593 | 0.923 | 0.234 |
| GLMnet | 0.742 | 0.982 | 0.077 | 0.879 | 0.389 |
| KNN | 0.584 | 0.974 | 0.011 | 0.870 | 0.059 |
| DT | 0.661 | 0.976 | 0.110 | 0.882 | 0.400 |
| RF ranger | 0.756 | 0.716 | 0.571 | 0.919 | 0.228 |
| SVM kernlab | 0.691 | 0.987 | 0.033 | 0.874 | 0.273 |
| LASSO | 0.740 | 0.882 | 0.308 | 0.897 | 0.277 |
| NB | 0.637 | 0.994 | 0.011 | 0.872 | 0.200 |
| AdaBoost | 0.765 | 0.987 | 0.022 | 0.873 | 0.200 |
| NNET | 0.617 | 0.981 | 0.033 | 0.873 | 0.200 |

AUC was calculated using predicted probabilities. Sensitivity, Specificity, PPV, and NPV were calculated based on the predicted class labels generated by the trained model. *ML*, machine learning; *IVIG*, intravenous immunoglobulin; *KD*, Kawasaki disease; *AUC*, area under the receiver operating characteristic curve; *PPV*, positive predictive value; *NPV*, negative predictive value; *RF*, random forest; *GBM*, gradient boosting machine; *GLMnet*, generalized linear model with elastic net regularization; *SVM*, support vector machine; *LASSO*, least absolute shrinkage and selection operator; *DT*, decision tree; *KNN*, K-nearest neighbor; *NB*, naïve Bayes; *Adaboost*, adaptive boosting; *NNET*, neural network; *RF ranger*, RF with ranger algorithm; *SVM kernlab*, SVM with kernlab algorithm.

**Supplementary Table 3 Model performance with different numbers of features.**

| Model | Feature numbers | AUC | Sensitivity | Specificity | PPV | NPV |
| --- | --- | --- | --- | --- | --- | --- |
| SVM | 32 | 0.742 | 0.798 | 0.524 | 0.913 | 0.260 |
|  | 26 | 0.767 | 0.826 | 0.545 | 0.919 | 0.299 |
|  | 20 | 0.753 | 0.782 | 0.535 | 0.913 | 0.250 |
|  | 14 | 0.756 | 0.748 | 0.622 | 0.924 | 0.254 |
|  | 8 | 0.782 | 0.735 | 0.721 | 0.940 | 0.274 |
|  | 2 | 0.736 | 0.645 | 0.776 | 0.943 | 0.233 |
| GBM | 32 | 0.604 | 0.901 | 0.253 | 0.891 | 0.274 |
|  | 26 | 0.619 | 0.874 | 0.286 | 0.893 | 0.250 |
|  | 20 | 0.564 | 0.787 | 0.297 | 0.884 | 0.170 |
|  | 14 | 0.570 | 0.795 | 0.253 | 0.879 | 0.153 |
|  | 8 | 0.538 | 0.706 | 0.341 | 0.879 | 0.146 |
|  | 2 | 0.581 | 0.572 | 0.560 | 0.898 | 0.161 |
| RF ranger | 32 | 0.718 | 0.934 | 0.176 | 0.885 | 0.281 |
|  | 26 | 0.733 | 0.935 | 0.209 | 0.889 | 0.322 |
|  | 20 | 0.717 | 0.927 | 0.165 | 0.883 | 0.250 |
|  | 14 | 0.719 | 0.939 | 0.198 | 0.888 | 0.321 |
|  | 8 | 0.745 | 0.947 | 0.187 | 0.888 | 0.340 |
|  | 2 | 0.690 | 0.758 | 0.462 | 0.905 | 0.219 |

AUC was calculated using predicted probabilities. Sensitivity, Specificity, PPV, and NPV were calculated based on the predicted class labels generated by the trained model. *SVM*, support vector machine; *GBM*, gradient boosting machine; *RF ranger*, random forest with ranger algorithm; *AUC*, area under the receiver operating characteristic curve; *PPV*, positive predictive value; *NPV*, negative predictive value.

**Supplementary Table 4 Comparison of IVIG-resistant and IVIG-responsive patients in the external validation and prospective cohorts.**

| **Characteristics** | **Fuzhou University Affiliated Provincial Hospital (May 2012 to May 2024)** | | | **Affiliated Hospital of Yangzhou University (January 2019 to December 2024)** | | | **Children’s Hospital of Soochow University**  **(July 2024 to December 2024)** | | |
| --- | --- | --- | --- | --- | --- | --- | --- | --- | --- |
|  | **IVIG-responsive (*n* = 401)** | **IVIG-resistant (*n* = 42)** | ***P*-value** | **IVIG-responsive (*n* = 170)** | **IVIG-resistant (*n* = 28)** | ***P*-value** | **IVIG-responsive (*n* = 217)** | **IVIG-resistant (*n* = 36)** | ***P*-value** |
| CALs | 38 (9.5) | 5 (11.9) | 0.817 | 39 (22.9) | 9 (32.1) | 0.292 | 25 (11.5) | 26 (72.2) | < 0.001 |
| CRP (mg/L) | 54.95 (28.47, 87.33) | 67.62 (38.45, 101.28) | 0.097 | 59.84 (32.23, 94.22) | 95.93 (57.89, 128.95) | 0.001 | 47.28 (26.59, 69.74) | 105.30 (69.02, 124.01) | < 0.001 |
| Age at presentation (mon) | 24.00 (15.00, 48.00) | 24.00 (13.00, 48.00) | 0.614 | 26.00 (15.00, 43.25) | 30.00 (15.75, 49.00) | 0.571 | 34.00 (21.50, 53.00) | 27.00 (19.00, 44.00) | 0.089 |
| NEU% | 53.00 (35.00, 71.00) | 68.00 (56.50, 83.00) | < 0.001 | 57.00 (41.80, 69.28) | 68.55 (55.60, 85.60) | 0.001 | 59.90 (46.35, 74.80) | 70.15 (54.25, 82.20) | 0.034 |
| WBC (×10^9^/L) | 11.70 (8.27, 16.07) | 12.20 (8.38, 18.41) | 0.613 | 11.43 (7.70, 15.28) | 12.95 (9.49, 16.33) | 0.148 | 11.40 (8.16, 15.28) | 14.23 (8.91, 18.35) | 0.058 |
| TC (mmol/L) | 3.29 (2.90, 3.75) | 3.26 (2.90, 3.80) | 0.588 | 3.60 (3.16, 4.10) | 3.28 (2.73, 3.58) | 0.004 | 3.83 (3.36, 4.27) | 3.36 (2.94, 4.11) | 0.041 |
| PLT (×10^9^/L) | 357.00 (271.50, 462.00) | 351.50 (283.25, 456.00) | 0.854 | 391.50 (292.25, 480.75) | 323.00 (187.50, 443.25) | 0.025 | 366.00 (291.00, 446.00) | 327.00 (286.25, 376.00) | 0.036 |
| ALB (g/L) | 37.50 (34.00, 40.00) | 35.10 (31.98, 39.03) | 0.049 | 40.10 (38.08, 41.90) | 37.95 (35.08, 39.65) | < 0.001 | 40.70 (38.90, 42.35) | 38.75 (36.30, 41.68) | 0.010 |

Median (interquartile range, IQR) is used for continuous variables, and number (%) for categorical variables. Categorical variables were compared using the chi-square test or Fisher’s exact test, and continuous variables using the Mann–Whitney *U* test. *P* < 0.05 is considered statistical significance. *IVIG*, intravenous immunoglobulin; *CALs*, coronary artery lesions; *CRP*, C-reactive protein; *NEU%*, neutrophil percentage; *WBC*, white blood cell; *TC*, total cholesterol; *PLT*, platelet; *ALB*, albumin.

**Supplementary Table 5 Comparison of SHAP-derived features included in the final SVM model between IVIG-responsive and IVIG-resistant patients.**

| **Characteristics** | **Overall (*n* = 2371)** | **IVIG-responsive (*n* = 2066)** | **IVIG-resistant (*n* = 305)** | ***P*-value** |
| --- | --- | --- | --- | --- |
| **Aged 0–5 mon** | | | | |
| Number | 165 | 138 | 27 |  |
| CALs | 71 (43.0) | 56 (40.6) | 15 (55.6) | 0.151 |
| CRP (mg/L) | 73.89 (37.61,106.11) | 70.02 (34.89, 106.02) | 84.62 (65.80, 112.79) | 0.051 |
| NEU% | 50.40 (32.20,60.55) | 47.70 (28.60, 59.33) | 57.00 (49.50, 66.80) | 0.016 |
| WBC (×10^9^/L) | 13.36 (9.50,16.16) | 13.05 (9.45, 15.70) | 15.19 (10.74, 20.77) | 0.110 |
| TC (mmol/L) | 3.18 (2.72,3.63) | 3.21 (2.81, 3.65) | 2.94 (2.54, 3.52) | 0.049 |
| PLT (×10^9^/L) | 466.00 (342.00,599.00) | 466.50 (345.50, 599.25) | 454.00 (325.00, 599.00) | 0.597 |
| ALB (g/L) | 40.20 (37.30,42.05) | 40.40 (37.63, 42.13) | 39.50 (35.80, 41.50) | 0.197 |
| **Aged 6–11 mon** | | | | |
| Number | 269 | 240 | 29 |  |
| CALs | 107 (39.8) | 89 (37.1) | 18 (62.1) | 0.019 |
| CRP (mg/L) | 64.06 (42.06,95.27) | 63.64 (41.99, 89.95) | 82.39 (47.12, 120.91) | 0.026 |
| NEU% | 46.50 (31.85,61.35) | 45.25 (31.78, 59.00) | 60.20 (34.05, 76.15) | 0.015 |
| WBC (×10^9^/L) | 13.90 (10.32,18.26) | 13.82 (10.30, 18.24) | 14.28 (10.10, 18.96) | 0.892 |
| TC (mmol/L) | 3.55 (3.06,4.06) | 3.55 (3.12, 4.07) | 3.36 (2.80, 3.99) | 0.204 |
| PLT (×10^9^/L) | 420.00 (341.50,535.50) | 425.50 (345.75, 540.75) | 362.00 (290.00, 516.00) | 0.082 |
| ALB (g/L) | 41.10 (38.50,43.00) | 41.30 (38.50, 43.10) | 40.60 (38.80, 41.55) | 0.226 |
| **Aged 1-2 y** |  |  |  |  |
| Number | 1075 | 912 | 163 |  |
| CALs | 309(28.7) | 221(24.2) | 88(54.0) | < 0.001 |
| CRP (mg/L) | 61.28(36.37,99.72) | 57.76 (32.18, 92.91) | 88.96 (55.98, 123.95) | < 0.001 |
| NEU% | 58.40(42.80,70.70) | 57.35 (40.70, 69.20) | 66.50 (53.70, 78.80) | < 0.001 |
| WBC (×10^9^/L) | 12.56(9.13,16.48) | 12.33 (8.85, 16.07) | 13.83 (10.36, 19.11) | < 0.001 |
| TC (mmol/L) | 3.70(3.22,4.19) | 3.73 (3.26, 4.23) | 3.50 (3.07, 3.97) | 0.001 |
| PLT (×10^9^/L) | 363.00(284.00,461.00) | 366.00 (290.00, 460.75) | 335.00 (246.00, 466.00) | 0.059 |
| ALB (g/L) | 40.10(37.60,42.20) | 40.20 (37.90, 42.30) | 39.20 (36.00, 41.70) | 0.001 |
| **Aged 3-4 y** |  |  |  |  |
| Number | 518 | 456 | 62 |  |
| CALs | 122(23.6) | 95(20.8) | 27(43.5) | < 0.001 |
| CRP (mg/L) | 64.62(35.45, 103.39) | 60.31 (32.38, 95.19) | 105.32 (70.63, 133.74) | < 0.001 |
| NEU% | 69.10(52.90, 81.00) | 67.50 (51.55, 79.88) | 76.15 (65.43, 87.23) | < 0.001 |
| WBC (×10^9^/L) | 11.58(8.07, 15.53) | 11.51 (7.83, 15.37) | 12.72 (9.49, 18.33) | 0.004 |
| TC (mmol/L) | 3.69(3.19, 4.15) | 3.75 (3.24, 4.21) | 3.33 (2.92, 3.90) | 0.001 |
| PLT (×10^9^/L) | 338.00(274.00, 414.25) | 346.00 (279.25, 423.75) | 301.00 (245.75, 362.50) | 0.001 |
| ALB (g/L) | 39.35(37.30, 41.10) | 39.50 (37.43, 41.10) | 38.35 (35.68, 40.23) | 0.020 |
| **Aged ≥ 5 years** |  |  |  |  |
| Number | 344 | 320 | 24 |  |
| CALs | 61(17.7) | 50(15.6) | 11(45.8) | 0.001 |
| CRP (mg/L) | 67.27(36.89,104.27) | 67.09 (36.89, 103.17) | 68.58 (37.16, 117.98) | 0.483 |
| NEU% | 73.95(56.43,83.90) | 74.00 (56.68, 83.88) | 68.55 (54.03, 89.83) | 0.817 |
| WBC (×10^9^/L) | 11.92(8.30,15.68) | 11.89 (8.30, 15.59) | 13.66 (7.79, 20.02) | 0.359 |
| TC (mmol/L) | 3.71(3.11,4.22) | 3.72 (3.15, 4.23) | 3.22 (2.83, 3.84) | 0.011 |
| PLT (×10^9^/L) | 340.50(264.00,435.25) | 340.50 (264.50, 433.00) | 345.50 (241.25, 465.00) | 0.975 |
| ALB (g/L) | 39.25(37.20,41.50) | 39.30 (37.20, 41.48) | 38.75 (35.05, 41.78) | 0.383 |

Median (interquartile range, IQR) is used for continuous variables, and number (%) for categorical variables. Categorical variables were compared using the chi-square test or Fisher’s exact test, and continuous variables using the Mann–Whitney *U* test. *P* < 0.05 is considered statistical significance.

*IVIG*, intravenous immunoglobulin; *CALs*, coronary artery lesions; *CRP*, C-reactive protein; *NEU%*, neutrophil percentage; *WBC*, white blood cell; *TC*, total cholesterol; *PLT*, platelet; *ALB*, albumin; *SHAP*, SHapley Additive exPlanations; *SVM*, support vector machine.

**Supplementary Table 6 Comparison of IVIG resistance prediction scoring systems.**

| **Prediction models** | **AUC** | **Sensitivity** | **Specificity** |
| --- | --- | --- | --- |
| Kobayashi (2006) | 0.655 | 0.452 | 0.774 |
| Egami (2006) | 0.653 | 0.347 | 0.817 |
| Sano (2007) | 0.672 | 0.630 | 0.636 |
| Formosa (2016) | 0.619 | 0.780 | 0.405 |
| Li (2019) | 0.674 | 0.593 | 0.657 |
| Our new scoring system | 0.782 | 0.735 | 0.721 |

Sensitivity and specificity were calculated at the optimal cutoff determined by the Youden index. *IVIG*, intravenous immunoglobulin; *AUC*, area under the receiver operating characteristic curve.

**Supplementary Table 7 Performance of ML models for IVIG resistance in KD after exclusion of CALs.**

| Model | AUC | Sensitivity | Specificity | PPV | NPV |
| --- | --- | --- | --- | --- | --- |
| RF | 0.660 | 0.987 | 0.022 | 0.873 | 0.200 |
| GBM | 0.690 | 0.987 | 0.022 | 0.873 | 0.200 |
| SVM | 0.721 | 0.885 | 0.297 | 0.895 | 0.276 |
| GLMnet | 0.739 | 0.722 | 0.615 | 0.927 | 0.246 |
| KNN | 0.537 | 0.989 | 0.011 | 0.872 | 0.125 |
| DT | 0.508 | 0.984 | 0.033 | 0.874 | 0.231 |
| RF ranger | 0.725 | 0.979 | 0.066 | 0.877 | 0.316 |
| SVM kernlab | 0.739 | 0.770 | 0.655 | 0.929 | 0.258 |
| LASSO | 0.740 | 0.727 | 0.615 | 0.928 | 0.249 |
| NB | 0.677 | 0.966 | 0.110 | 0.881 | 0.323 |
| AdaBoost | 0.670 | 0.989 | 0.044 | 0.876 | 0.364 |
| NNET | 0.545 | 0.956 | 0.033 | 0.871 | 0.100 |

AUC was calculated using predicted probabilities. Sensitivity, Specificity, PPV, and NPV were calculated based on the predicted class labels generated by the trained model. *ML*, machine learning; *IVIG*, intravenous immunoglobulin; *KD*, Kawasaki disease; *CALs,* coronary artery lesions; *AUC*, area under the receiver operating characteristic curve; *PPV*, positive predictive value; *NPV*, negative predictive value; *RF*, random forest; *GBM*, gradient boosting machine; *GLMnet*, generalized linear model with elastic net regularization; *SVM*, support vector machine; *LASSO*, least absolute shrinkage and selection operator; *DT*, decision tree; *KNN*, K-nearest neighbor; *NB*, naïve Bayes; *Adaboost*, adaptive boosting; *NNET*, neural network; *RF ranger*, RF with ranger algorithm; *SVM kernlab*, SVM with kernlab algorithm.

**Supplementary Table 8 SVM kernlab model performance with different numbers of features after exclusion of CALs.**

| Feature numbers | AUC | Sensitivity | Specificity | PPV | NPV |
| --- | --- | --- | --- | --- | --- |
| 35 | 0.730 | 0.755 | 0.600 | 0.918 | 0.231 |
| 32 | 0.740 | 0.718 | 0.710 | 0.934 | 0.240 |
| 29 | 0.734 | 0.747 | 0.644 | 0.925 | 0.239 |
| 26 | 0.751 | 0.802 | 0.578 | 0.919 | 0.258 |
| 23 | 0.726 | 0.751 | 0.633 | 0.923 | 0.238 |
| 20 | 0.740 | 0.778 | 0.611 | 0.922 | 0.250 |
| 17 | 0.728 | 0.759 | 0.633 | 0.924 | 0.243 |
| 14 | 0.728 | 0.725 | 0.666 | 0.927 | 0.232 |
| 11 | 0.723 | 0.718 | 0.677 | 0.928 | 0.231 |
| 8 | 0.719 | 0.739 | 0.655 | 0.926 | 0.237 |
| 5 | 0.762 | 0.721 | 0.721 | 0.937 | 0.245 |
| 2 | 0.704 | 0.757 | 0.578 | 0.915 | 0.225 |

AUC was calculated using predicted probabilities. Sensitivity, Specificity, PPV, and NPV were calculated based on the predicted class labels generated by the trained model. *SVM kernlab*, support vector machine with kernlab algorithm; *CALs*, coronary artery lesions; *AUC*, area under the receiver operating characteristic curve; *PPV*, positive predictive value; *NPV*, negative predictive value.

**Supplementary Table 9** **TRIPOD+AI checklist for reporting the development and evaluation of the prediction model.**

| **Section / Topic item** | | **Development / evaluation**^1^ | **Checklist item** | **Reported**  **on page** |
| --- | --- | --- | --- | --- |
| **TITLE** | | | |  |
| *Title* | 1 | D;E | Identify the study as developing or evaluating the performance of a multivariable prediction model, the target population, and the outcome to be predicted | Title page |
| **ABSTRACT** | | | |  |
| *Abstract* | 2 | D;E | See TRIPOD+AI for Abstracts checklist | Page 1 |
| **INTRODUCTION** | | | |  |
| *Background* | 3a | D;E | Explain the healthcare context (including whether diagnostic or prognostic) and rationale for developing or evaluating the prediction model, including references to existing models | Page 3 |
|  | 3b | D;E | Describe the target population and the intended purpose of the prediction model in the context of the care pathway, including its intended users (e.g., healthcare professionals, patients, public) | Page 4 |
|  | 3c | D;E | Describe any known health inequalities between sociodemographic groups | Not reported |
| *Objectives* | 4 | D;E | Specify the study objectives, including whether the study describes the development or validation of a prediction model (or both) | Page 4 |
| **METHODS** | | | |  |
| *Data* | 5a | D;E | Describe the sources of data separately for the development and evaluation datasets (e.g., randomised trial, cohort, routine care or registry data), the rationale for using these data, and representativeness of the data | Page 4 |
|  | 5b | D;E | Specify the dates of the collected participant data, including start and end of participant accrual; and, if applicable, end of follow-up | Page 4 |
| *Participants* | 6a | D;E | Specify key elements of the study setting (e.g., primary care, secondary care, general population) including the number and location of centres | Page 4 |
|  | 6b | D;E | Describe the eligibility criteria for study participants | Supplementary Materials |
|  | 6c | D;E | Give details of any treatments received, and how they were handled during model development or evaluation, if relevant | Page 4 |
| *Data preparation* | 7 | D;E | Describe any data pre-processing and quality checking, including whether this was similar across relevant sociodemographic groups | Supplementary Materials |
| *Outcome* | 8a | D;E | Clearly define the outcome that is being predicted and the time horizon, including how and when  assessed, the rationale for choosing this outcome, and whether the method of outcome assessment is consistent across sociodemographic groups | Page 4 |
|  | 8b | D;E | If outcome assessment requires subjective interpretation, describe the qualifications and demographic characteristics of the outcome assessors | Supplementary Materials |
|  | 8c | D;E | Report any actions to blind assessment of the outcome to be predicted | Not reported |
| *Predictors* | 9a | D | Describe the choice of initial predictors (e.g., literature, previous models, all available predictors) and any pre-selection of predictors before model building | Page 5, Supplementary Materials |
|  | 9b | D;E | Clearly define all predictors, including how and when they were measured (and any actions to blind assessment of predictors for the outcome and other predictors) | Page 4, |
|  | 9c | D;E | If predictor measurement requires subjective interpretation, describe the qualifications and demographic characteristics of the predictor assessors | Supplementary Materials |
| *Sample size* | 10 | D;E | Explain how the study size was arrived at (separately for development and evaluation), and justify that the study size was sufficient to answer the research question. Include details of any sample size  calculation | Not reported |
| *Missing data* | 11 | D;E | Describe how missing data were handled. Provide reasons for omitting any data | Page 5, Supplementary Materials |
| *Analytical methods* | 12a | D | Describe how the data were used (e.g., for development and evaluation of model performance) in the analysis, including whether the data were partitioned, considering any sample size requirements | Page 5 |
|  | 12b | D | Depending on the type of model, describe how predictors were handled in the analyses (functional form, rescaling, transformation, or any standardisation). | Supplementary Materials |
|  | 12c | D | Specify the type of model, rationale^2^, all model-building steps, including any hyperparameter tuning, and method for internal validation | Page 5, Supplementary Materials |
|  | 12d | D;E | Describe if and how any heterogeneity in estimates of model parameter values and model performance was handled and quantified across clusters (e.g., hospitals, countries). See TRIPOD-Cluster for  additional considerations^3^ | Page 5 |
|  | 12e | D;E | Specify all measures and plots used (and their rationale) to evaluate model performance (e.g., discrimination, calibration, clinical utility) and, if relevant, to compare multiple models | Page 5, Supplementary Materials |
|  | 12f | E | Describe any model updating (e.g., recalibration) arising from the model evaluation, either overall or for particular sociodemographic groups or settings | Not reported |
|  | 12g | E | For model evaluation, describe how the model predictions were calculated (e.g., formula, code, object, application programming interface) | Page 9 |
| *Class imbalance* | 13 | D;E | If class imbalance methods were used, state why and how this was done, and any subsequent methods to recalibrate the model or the model predictions | Not reported |
| *Fairness* | 14 | D;E | Describe any approaches that were used to address model fairness and their rationale | Not reported |
| *Model output* | 15 | D | Specify the output of the prediction model (e.g., probabilities, classification). Provide details and rationale for any classification and how the thresholds were identified | Page 9-10 |
| *Training versus evaluation* | 16 | D;E | Identify any differences between the development and evaluation data in healthcare setting, eligibility criteria, outcome, and predictors | Page 14-15 |
| *Ethical approval* | 17 | D;E | Name the institutional research board or ethics committee that approved the study and describe the participant-informed consent or the ethics committee waiver of informed consent | Page 4 |
| **OPEN SCIENCE** | | | | |
| *Funding* | 18a | D;E | Give the source of funding and the role of the funders for the present study | Title page |
| *Conflicts of interest* | 18b | D;E | Declare any conflicts of interest and financial disclosures for all authors | Title page |
| *Protocol* | 18c | D;E | Indicate where the study protocol can be accessed or state that a protocol was not prepared | Not reported |
| *Registration* | 18d | D;E | Provide registration information for the study, including register name and registration number, or state that the study was not registered | Not reported |
| *Data sharing* | 18e | D;E | Provide details of the availability of the study data | Title page |
| *Code sharing* | 18f | D;E | Provide details of the availability of the analytical code^4^ | Not reported |
| **PATIENT & PUBLIC INVOLVEMENT** | | | | |
| *Patient & Public Involvement* | 19 | D;E | Provide details of any patient and public involvement during the design, conduct, reporting,  interpretation, or dissemination of the study or state no involvement. | Not reported |
| **RESULTS** | | | | |
| *Participants* | 20a | D;E | Describe the flow of participants through the study, including the number of participants with and  without the outcome and, if applicable, a summary of the follow-up time. A diagram may be helpful. | Figure1 |
|  | 20b | D;E | Report the characteristics overall and, where applicable, for each data source or setting, including the key dates, key predictors (including demographics), treatments received, sample size, number of  outcome events, follow-up time, and amount of missing data. A table may be helpful. Report any differences across key demographic groups. | Baseline table |
|  | 20c | E | For model evaluation, show a comparison with the development data of the distribution of important predictors (demographics, predictors, and outcome). | Table1, Supplementary Table 4 |
| *Model development* | 21 | D;E | Specify the number of participants and outcome events in each analysis (e.g., for model development, hyperparameter tuning, model evaluation) | Page 7 |
| *Model*  *specification* | 22 | D | Provide details of the full prediction model (e.g., formula, code, object, application programming interface) to allow predictions in new individuals and to enable third-party evaluation and  implementation, including any restrictions to access or re-use (e.g., freely available, proprietary)^5^ | Page 9 |
| *Model*  *performance* | 23a | D;E | Report model performance estimates with confidence intervals, including for any key subgroups (e.g., sociodemographic). Consider plots to aid presentation. | Page 8-10 |
|  | 23b | D;E | If examined, report results of any heterogeneity in model performance across clusters. See TRIPOD Cluster for additional details^3^. | Not reported |
| *Model updating* | 24 | E | Report the results from any model updating, including the updated model and subsequent performance | Not reported |
| **DISCUSSION** | | | | |
| *Interpretation* | 25 | D;E | Give an overall interpretation of the main results, including issues of fairness in the context of the objectives and previous studies | Page 12-17 |
| *Limitations* | 26 | D;E | Discuss any limitations of the study (such as a non-representative sample, sample size, overfitting, missing data) and their effects on any biases, statistical uncertainty, and generalizability | Page 17 |
| *Usability of the*  *model in the*  *context of current care* | 27a | D | Describe how poor quality or unavailable input data (e.g., predictor values) should be assessed and handled when implementing the prediction model | Not reported |
|  | 27b | D | Specify whether users will be required to interact in the handling of the input data or use of the model, and what level of expertise is required of users | Page 9 |
|  | 27c | D;E | Discuss any next steps for future research, with a specific view to applicability and generalizability of the model | Page 17 |

D represents items relevant only to the development of a prediction model; E represents items relating solely to the evaluation of a prediction model; D; E=items applicable to both the development and evaluation of a prediction model. Reproduced from “TRIPOD+AI statement: updated guidance for reporting clinical prediction models that use regression or machine learning methods” by Collins GS, et al. [16] with permitted copyright.

**Supplementary Table 10 PROBAST+AI-based assessment of methodological quality, risk of bias, and applicability of the prediction model.**

| **Model development: Signaling questions** | **Assessment (Y/PY/PN/N/NI/NA)** | **Model evaluation: Signaling questions** | **Assessment (Y/PY/PN/N/NI/NA)** |
| --- | --- | --- | --- |
| **Participants and data sources** | | | |
| 1.1 Were appropriate data sources used? | Y | 1.1 Were appropriate data sources used? | Y |
| 1.2 Was an appropriate study design used? | N | 1.2 Was an appropriate study design used? | N |
| 1.3 Did the inclusions and exclusions of study participants result in a representative dataset? | Y | 1.3 Did the inclusions and exclusions of study participants result in a representative dataset? | Y |
| Quality: Concern regarding quality of selection of participants and data sources | High Risk | Quality: Concern regarding quality of selection of participants and data sources | High Risk |
| Applicability: Concern that the data of the included participants do not match the review question or the assessor’s intended use of the prediction model | Low Risk | Applicability: Concern that the data of the included participants do not match the review question or the assessor’s intended use of the prediction model | Low Risk |
| **Predictors** | | | |
| 2.1 Were predictors defined and assessed in a similar way for all participants? | Y | 2.1 Were predictors defined and assessed in a similar way for all participants? | Y |
| 2.2 Was any preprocessing of predictors similar for all participants? | Y | 2.2 Was any preprocessing of predictors similar for all participants? | Y |
| 2.3 Were predictor assessments made without knowledge of outcome data? | N | 2.3 Were predictor assessments made without knowledge of outcome data? | N |
| 2.4 Were the predictors included in the model available at the time the model was intended to be used? | Y | 2.4 Were the predictors included in the model available at the time the model was intended to be used? | Y |
| Quality: Concern regarding the quality of the predictors or their assessment | High Risk | Quality: Concern regarding the quality of the predictors or their assessment | High Risk |
| Applicability: Concern that the definition, preprocessing, assessment, or timing of assessment of the predictors in the model do not match the review question or the assessor’s intended use | Low Risk | Applicability: Concern that the definition, preprocessing, assessment, or timing of assessment of the predictors in the model do not match the review question or the assessor’s intended use | Low Risk |
| **Outcomes** | |  |  |
| 3.1 Were outcomes defined and assessed appropriately? | Y | 3.1 Were outcomes defined and assessed appropriately? | Y |
| 3.2 Were outcomes defined and assessed in a similar way for all participants? | Y | 3.2 Were outcomes defined and assessed in a similar way for all participants? | Y |
| 3.3 Were outcome assessments made without use or knowledge of predictor data? | Y | 3.3 Were outcome assessments made without use or knowledge of predictor data? | Y |
| 3.4 Was the time interval between predictor assessment and outcome assessment appropriate? | Y | 3.4 Was the time interval between predictor assessment and outcome assessment appropriate? | Y |
| Quality: Concern regarding quality of the outcome or its determination | Low Risk | Quality: Concern regarding quality of the outcome or its determination | Low Risk |
| Applicability: Concern that the outcome, its definition, assessment, or timing of assessment do not match the review question or the assessor’s intended use | Low Risk | Applicability: Concern that the outcome, its definition, assessment, or timing of assessment do not match the review question or the assessor’s intended use | Low Risk |
| **Analyses** | | | |
| 4.1 Was there evidence that the sample size was reasonable? | N | 4.1 Was model evaluation based on only apparent performance avoided? | N |
| 4.2 Were continuous and categorical predictors handled appropriately? | Y | 4.2 Was there evidence that the sample size was reasonable? | Y |
| 4.3 Were participants with missing or censored data handled appropriately in the analysis? | Y | 4.3 Were participants with missing or censored data handled appropriately in the analysis? | Y |
| 4.4 If methods to address class imbalance were used, was the model or the model predictions recalibrated? | NA | 4.4 If methods to address class imbalance were used, was the evaluation done in a dataset without correction for imbalance? | NA |
| 4.5 Were methods used to address potential model overfitting? | Y | 4.5 If data splitting was done to create training and test datasets, was there evidence that data leakage was avoided? | Y |
|  |  | 4.6 If resampling methods were used to evaluate model performance, were all model development steps replicated in the resampling process? | Y |
|  |  | 4.7 Was the predictive performance of the model evaluated appropriately—for example, calibration, discrimination, and net benefit? | Y |
| Quality: Concern regarding quality of the analysis | High Risk | Risk of bias: Risk of bias introduced by the analysis | High Risk |

Methodological quality, risk of bias, and applicability were classified as low, high, or unclear. *Y*, yes; *PY*, probably yes; *PN*, probably no; *N*, no; *NI*, no information; *NA*, not applicable. Reproduced from “PROBAST+AI: an updated quality, risk of bias, and applicability assessment tool for prediction model studies using regression or artificial intelligence methods” by Moons KGM, et al. [17] with permitted copyright.

**Supplementary figures and figure legends**

**Supplementary Fig. 1 Spearman correlation heatmap of retained features.**
Displays pairwise correlations among the 38 features selected for model construction. Variables with >50% missing data were excluded, and variables with high collinearity (*r* > 0.6) were removed. Red and blue colours represent positive and negative correlations, respectively, and dendrogram branches indicate hierarchical clustering of variables. *IVIGR* intravenous immunoglobulin resistance; *Hb* hemoglobin; *TC* total cholesterol; *ALB* albumin; *CK* creatine kinase; *CKMB* creatine kinase-MB; *Mg* magnesium; *K* potassium; *iKD* incomplete Kawasaki disease; *Na* sodium; *Cl* chloride; *EOS%* eosinophil percentage; *PLT* platelet; *MONO%* monocyte percentage; *LYMPH%* lymphocyte percentage; *CR* creatinine; *BUN* blood urea nitrogen; *NEU%* neutrophil percentage; *WBC* white blood cell; *LDH* lactate dehydrogenase; *CRP* C-reactive protein; *ALT* alanine aminotransferase; *GGT* γ-glutamyl transpeptidase; *AST* aspartate aminotransferase; *ALP* alkaline phosphatase; *Ca* calcium; *CALs* coronary artery lesions; *TG* triglycerides; *GLU* glucose; *UWBC* urinary white blood cell; *Perianal desq.*, perianal desquamation; *Cervical LAP*, cervical lymphadenopathy; *Conjunctival inj.*, conjunctival injection.

**Supplementary Fig. 2 Top 10 predictive features ranked by SHAP value across three ML models.**

**a** SHAP summary plot for the SVM model, **b** SHAP summary plot for the GBM model, **c** SHAP summary plot for the RF ranger model. Red and blue colours indicate high and low feature values, respectively, and the horizontal distribution of points reflects the impact of each feature on model output. *SHAP* SHapley Additive exPlanations; *GBM* gradient boosting machine; *SVM* support vector machine; *RF ranger*, random forest with ranger algorithm; *CALs* coronary artery lesions; *CRP* C-reactive protein; *NEU%* neutrophil percentage; *WBC* white blood cell; *TC* total cholesterol; *PLT* platelet; *ALB* albumin; *Na* sodium; *CK* creatine kinase; *CR* creatinine; *GGT* γ-glutamyl transpeptidase; *TG* triglycerides; *UWBC* urinary white blood cell; *AST* aspartate aminotransferase; *EOS%* eosinophil percentage.

**Supplementary Fig. 3 Changes in sensitivity and specificity during stepwise feature elimination.**
**a** Changes in sensitivity during stepwise feature elimination, **b** Changes in specificity during stepwise feature elimination, with the vertical dashed line indicating the final eight-feature model. *GBM* gradient boosting machine; *RF ranger*, random forest with ranger algorithm; *SVM* support vector machine.

**Supplementary Fig. 4 Local interpretability of IVIG resistance predictions using SHAP.**

SHAP value plots for four representative patients. Red and blue bars indicate positive and negative contributions to IVIG resistance risk, respectively, and bar length reflects the magnitude of feature contribution. f(x) represents the individualized predicted probability of IVIG resistance generated by the web-based prediction tool for each patient. *SHAP* SHapley Additive exPlanations; *CALs* coronary artery lesions; *CRP* C-reactive protein; *NEU%* neutrophil percentage; *WBC* white blood cell; *TC* total cholesterol; *PLT* platelet; *ALB* albumin.

**Supplementary Fig. 5 Global interaction strength of predictors in the final SVM model.**

Bar length represents the overall interaction strength of each predictor in the model. *SVM* support vector machine; *CALs* coronary artery lesions; *CRP* C-reactive protein; *NEU%* neutrophil percentage; *WBC* white blood cell; *TC* total cholesterol; *PLT* platelet; *ALB* albumin.

**Supplementary Fig. 6 Performance of 12 machine learning models for predicting IVIG resistance after exclusion of CALs.**
**a** ROC curves of all evaluated models, **b** Changes in AUC, sensitivity, and specificity during stepwise feature elimination, with the vertical dashed line indicating the final five-feature model. *IVIGR* intravenous immunoglobulin resistance; *CALs* coronary artery lesions; *ROC* receiver operating characteristic; *AUC* area under the ROC curve; *RF* random forest; *GBM* gradient boosting machine; *GLMnet* generalized linear model with elastic net regularization; *SVM* support vector machine; *LASSO* least absolute shrinkage and selection operator; *DT* decision tree; *KNN* K-nearest neighbor; *NB* naïve Bayes; *Adaboost* adaptive boosting; *NNET* neural network; *RF ranger*, RF with ranger algorithm; *SVM kernlab*, SVM with kernlab algorithm.

**Supplementary Fig. 7 Identification of predictive factors using LASSO and GLMnet regression after exclusion of CALs.**
**a** LASSO regression, **b** GLMnet regression. Red dots represent the average MSE values, error bars indicate standard errors, and numbers on the upper axis indicate the number of retained variables at each λ value. The vertical dotted lines indicate λ_min and λ_1se. *LASSO* least absolute shrinkage and selection operator; *GLMnet* generalized linear model with elastic net regularization; *CALs* coronary artery lesions; *MSE* mean-squared error.

**Supplementary Fig. 8 SHAP analysis of key predictors in the SVM kernlab model after exclusion of CALs.**
**a** Ranking of the top 10 predictive features based on average SHAP values, **b–f** Scatterplots of SHAP values versus feature values for the five key predictors in the final SVM kernlab model. The y-axis represents SHAP values, and the x-axis represents feature values. SHAP values >0 indicate positive contributions to IVIG resistance prediction. The red dashed lines represent fitted trend lines between feature values and SHAP values. *SHAP* SHapley Additive exPlanations; *SVM kernlab*, support vector machine with kernlab algorithm; *CALs* coronary artery lesions; *CRP* C-reactive protein; *LYMPH%* lymphocyte percentage; *EOS%* eosinophil percentage; *TC* total cholesterol; *NEU%* neutrophil percentage; *Cl* chloride; *WBC* white blood cell; *IVIGR* intravenous immunoglobulin resistance.

**Supplementary Fig. 9 Local interpretability of IVIG resistance predictions using SHAP after exclusion of CALs.**
SHAP value plots for four representative patients. Red and blue bars indicate positive and negative contributions to IVIG resistance risk, respectively, and bar length reflects the magnitude of feature contribution. *SHAP* SHapley Additive exPlanations; *IVIG* intravenous immunoglobulin; *CALs* coronary artery lesions; *CRP* C-reactive protein; *LYMPH%* lymphocyte percentage; *EOS%* eosinophil percentage.

**Supplementary Fig. 10 External and prospective validation of the final SVM kernlab model after exclusion of CALs.
a** ROC curve for external validation using the Fuzhou cohort, **b** ROC curve for prospective validation using the Suzhou cohort. The corresponding AUC, sensitivity, and specificity are shown in each panel. The blue solid lines represent the ROC curves of the final SVM kernlab model across different prediction thresholds, and the gray diagonal lines represent the no-discrimination reference line corresponding to random classification (AUC = 0.5). Curves closer to the upper-left corner indicate better discrimination. *IVIGR* intravenous immunoglobulin resistance; *CALs* coronary artery lesions; *SVM kernlab*, support vector machine with kernlab algorithm; *ROC* receiver operating characteristic; *AUC* area under the ROC curve.

**Supplementary Fig.11 Calibration and decision curve analyses of the SVM kernlab model after exclusion of CALs in different validation cohorts.**
**a** Calibration curves for internal validation, **b** External validation (Fuzhou cohort), **c** Prospective validation (Suzhou cohort). In a–c, the diagonal black lines represent ideal calibration, and the blue and red lines represent the apparent and bias-corrected curves, respectively. **d** DCA for internal validation, **e** External validation (Fuzhou cohort), **f** Prospective validation (Suzhou cohort). In d–f, the red, blue, and black lines represent the model, treat-all, and treat-none strategies, respectively. *SVM kernlab*, support vector machine with kernlab algorithm; *CALs* coronary artery lesions; *DCA* decision curve analysis.

**Reference:**

1.	McCrindle BW, Rowley AH, Newburger JW, Burns JC, Bolger AF, Gewitz M, et al. Diagnosis, treatment, and long-term management of Kawasaki disease: a scientific statement for health professionals from the American Heart Association. Circulation. 2017;135:e927-e99. doi:10.1161/cir.0000000000000484

2.	Jone PN, Tremoulet A, Choueiter N, Dominguez SR, Harahsheh AS, Mitani Y, et al. Update on diagnosis and management of Kawasaki disease: a scientific statement from the American Heart Association. Circulation. 2024;150:e481-e500. doi:10.1161/cir.0000000000001295

3.	Huang H, Jiang J, Shi X, Qin J, Dong J, Xu L, et al. Nomogram to predict risk of resistance to intravenous immunoglobulin in children hospitalized with Kawasaki disease in Eastern China. Ann Med. 2022;54:442-53. doi:10.1080/07853890.2022.2031273

4.	Hsu WH, Ko AT, Weng CS, Chang CL, Jan YT, Lin JB, et al. Explainable machine learning model for predicting skeletal muscle loss during surgery and adjuvant chemotherapy in ovarian cancer. J Cachexia Sarcopenia Muscle. 2023;14:2044-53. doi:10.1002/jcsm.13282

5.	Li J, Liu S, Hu Y, Zhu L, Mao Y, Liu J. Predicting mortality in intensive care unit patients with heart failure using an interpretable machine learning model: retrospective cohort study. J Med Internet Res. 2022;24:e38082. doi:10.2196/38082

6.	Jiang W, Zhang Y, Weng J, Song L, Liu S, Li X, et al. Explainable machine learning model for predicting persistent sepsis-associated acute kidney injury: development and validation study. J Med Internet Res. 2025;27:e62932. doi:10.2196/62932

7.	Meiseles A, Paley D, Ziv M, Hadid Y, Rokach L, Tadmor T. Explainable machine learning for chronic lymphocytic leukemia treatment prediction using only inexpensive tests. Comput Biol Med. 2022;145:105490. doi:10.1016/j.compbiomed.2022.105490

8.	Elhazmi A, Al-Omari A, Sallam H, Mufti HN, Rabie AA, Alshahrani M, et al. Machine learning decision tree algorithm role for predicting mortality in critically ill adult COVID-19 patients admitted to the ICU. J Infect Public Health. 2022;15:826-34. doi:10.1016/j.jiph.2022.06.008

9.	Siddalingappa R, Kanagaraj S. K-nearest-neighbor algorithm to predict the survival time and classification of various stages of oral cancer: a machine learning approach. F1000Res. 2022;11:70. doi:10.12688/f1000research.75469.2

10.	Barreñada L, Dhiman P, Timmerman D, Boulesteix AL, Van Calster B. Understanding overfitting in random forest for probability estimation: a visualization and simulation study. Diagn Progn Res. 2024;8:14. doi:10.1186/s41512-024-00177-1

11.	Karatzoglou A, Smola A, Hornik K, Zeileis A. kernlab - An S4 Package for Kernel Methods in R. J Stat Softw. 2004;11:1-20. doi:10.18637/jss.v011.i09

12.	Wang P, Fang E, Zhao X, Feng J. Nomogram for soiling prediction in postsurgery hirschsprung children: a retrospective study. Int J Surg. 2024;110:1627-36. doi:10.1097/js9.0000000000000993

13.	Zuo D, Yang L, Jin Y, Qi H, Liu Y, Ren L, et al. Machine learning-based models for the prediction of breast cancer recurrence risk. BMC Med Inform Decis Mak. 2023;23:276. doi:10.1186/s12911-023-02377-z

14.	Zhang H, Ma JX, Liu CT, Ren JX, Ding L. Development and evaluation of in silico prediction model for drug-induced respiratory toxicity by using naïve Bayes classifier method. Food Chem Toxicol. 2018;121:593-603. doi:10.1016/j.fct.2018.09.051

15.	Kriegeskorte N, Golan T. Neural network models and deep learning. Curr Biol. 2019;29:R231-6. doi:10.1016/j.cub.2019.02.034

16.	Collins GS, Moons KGM, Dhiman P, Riley RD, Beam AL, Van Calster B, et al. TRIPOD+AI statement: updated guidance for reporting clinical prediction models that use regression or machine learning methods. BMJ. 2024;385:e078378. doi:10.1136/bmj-2023-078378

17.	Moons KGM, Damen JAA, Kaul T, Hooft L, Andaur Navarro C, Dhiman P, et al. PROBAST+AI: an updated quality, risk of bias, and applicability assessment tool for prediction models using regression or artificial intelligence methods. BMJ. 2025;388:e082505. doi:10.1136/bmj-2024-082505
